# Cell fate roadmap of human primed-to-naive transition reveals preimplantation cell lineage signatures

**DOI:** 10.1038/s41467-022-30924-1

**Published:** 2022-06-07

**Authors:** Yan Bi, Zhifen Tu, Jianfeng Zhou, Xuehao Zhu, Hong Wang, Shaorong Gao, Yixuan Wang

**Affiliations:** 1 Shanghai Key Laboratory of Maternal and Fetal Medicine, Clinical and Translational Research Center of Shanghai First Maternity and Infant Hospital, School of Life Sciences and Technology, Tongji University, 200092 Shanghai, China; 2 Translational Medical Center for Stem Cell Therapy & Institute for Regenerative Medicine, Shanghai East Hospital, School of Life Sciences and Technology, Tongji University, 200120 Shanghai, China; 3 Frontier Science Center for Stem Cell Research, Tongji University, 200092 Shanghai, China

**Keywords:** Embryonic stem cells, Reprogramming, Pluripotency

## Abstract

Human naive pluripotent stem cells offer a unique window into early embryogenesis studies. Recent studies have reported several strategies to obtain cells in the naive state. However, cell fate transitions and the underlying mechanisms remain poorly understood. Here, by a dual fluorescent reporter system, we depict the cell fate dynamics from primed state toward naive pluripotency with ALPG activation followed by the activation of OCT4-distal enhancer. Integration of transcription profiles and the chromatin accessibility landscape reveals the appearance of primitive endoderm and trophectoderm signatures in the transitioning subpopulations, with the capacities for derivation of extra-embryonic endoderm and trophoblast stem cell lines, respectively. Furthermore, despite different fluorescent dynamics, all transitioning intermediates are capable of reaching the naive state with prolonged induction, showing their developmental plasticity and potential. Overall, our study describes a global cell roadmap toward naive pluripotency and provides hints for embryo modeling-related studies.

## Abstract (web summary)

Cell fate dynamics during human naïve pluripotency establishment remain poorly understood. Here, Bi et al. depict a high-resolution cell roadmap of the primed-to-naïve pluripotency transition, providing hints for embryo modeling-related studies.

## Introduction

Human naive pluripotent stem cells (PSCs) capture the ground pluripotent state corresponding to the preimplantation epiblast^1–4^ and exhibit more plasticity and unbiased differentiation potential than conventional PSCs in the primed pluripotent state^5–7^, thus providing an inexhaustible model for developmental studies and therapeutic applications. Important breakthroughs in culture system optimization have allowed the development of several strategies for achieving naive pluripotency. Naive PSCs can be generated by direct derivation from preimplantation embryos, reprogramming of somatic cells, or transitioning of conventional PSCs in the primed state^8–20^. Several molecular events during the establishment of naive pluripotency have been reported: The surface marker SSEA4 disappears during the derivation of naive PSCs directly from human preimplantation blastocysts or from PSCs in the primed state^9^; The activity of OCT4 enhancer swiches from proximal enhancer (PE) to distal enhancer during the transition of cells from primed state to naive state^12^; The expression of ALPG (also known as ALPPL2) is acquired during the establishment of naive pluripotency from cells at either primed state or somatic state^21^. Moreover, a recent study depicted a high-resolution roadmap for naive reprogramming process from somatic cells, showing the molecular reprogramming trajectories with trophectoderm (TE) lineage-specific signatures^22^. Although the molecular criteria for distinguishing naive and primed pluripotency have been systematically defined^9,23–27^, neither detailed molecular events nor subpopulation dynamics have been described during the primed-to-naive transition process.

In this study, to precisely monitor naive pluripotency establishment from cells in the primed state, we constructed a dual fluorescent reporter system composed of ALPG-promoter-RFP and OCT4-ΔPE-GFP, and traced the fluorescence dynamics during the process. Transcriptional profiling by both bulk and single cell RNA-seq (scRNA-seq) analyses shows a transitioning trajectory toward naive pluripotency, with ALPG activation followed by the activation of OCT4-distal enhancer. Integrative analysis with chromatin accessibility dynamics (CAD) indicates that primitive endoderm (PrE) signatures and TE signatures emerge successively in the transitioning subpopulations during the primed-to-naive induction process, and the intermediates with strong PrE or TE signatures enable the generation of extra-embryonic endoderm cell lines or trophoblast stem cell (TSC) lines, respectively. Furthermore, despite different fluorescent dynamics, all transitioning intermediate cells are capable of reaching a naive pluripotent state with prolonged induction by 5iLAF culture system, showing their developmental plasticity and potential. Overall, our study describes a high-resolution cell roadmap toward naive pluripotency, and provides valuable sources for human blastocyst modeling and early embryogenesis studies.

## Results

### Dual reporter system to monitor fluorescent dynamics during the primed-to-naive transition process

**Fig. 1:**
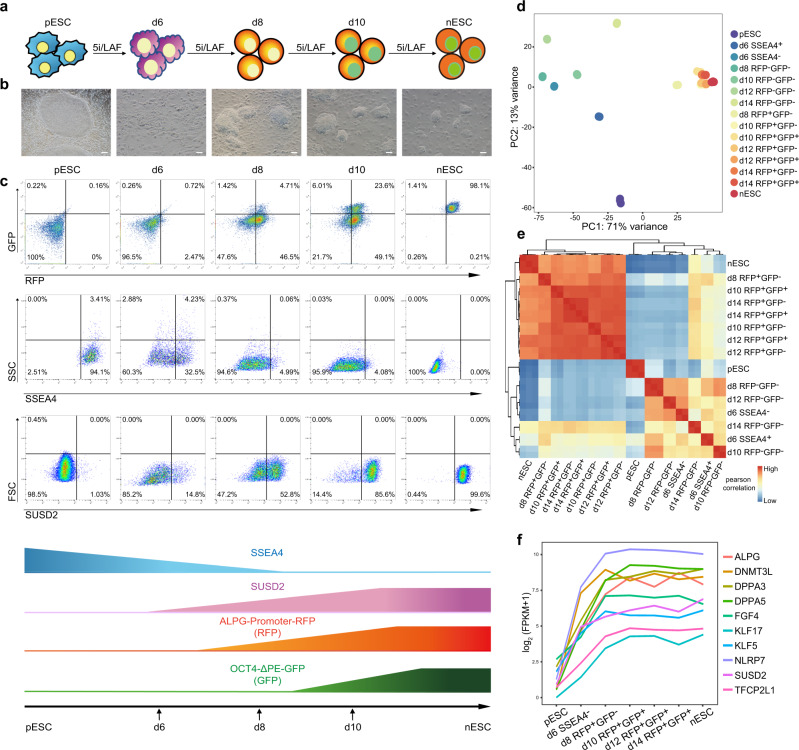
Transcriptional roadmaps for intermediates with fluorescence dynamics during the primed-to-naive transition process. **a** Schematic representation of the primed-to-naive transition using 5iLAF culture conditions. **b** Morphological changes of cells during the primed-to-naive transition. Scale bars, 50 μm. Representative images from *n* = 5. **c** Dynamics of SSEA4, SUSD2, ALPG-promoter-RFP^21^, and OCT4-ΔPE-GFP^12^ signals during the primed-to-naive transition process as determined by flow cytometry analysis. **d** PCA analysis of the bulk RNA-seq datasets collected from the primed-to-naive transition process. *n* ≥ 2. **e** Heatmap to indicate the Pearson correlation coefficients among bulk RNA-seq datasets. **f** Line plots showing the dynamics of representative naive-specific gene expression during the primed-to-naive transition. Source data are provided as a Source Data file.

To precisely investigate the dynamics during the primed-to-naive transition, we constructed a dual fluorescent reporter system. One was ALPG-promoter-RFP (hereafter mentioned as RFP), in which RFP was fused to the DNA sequences 3 kb upstream of ALPG transcription start site (TSS)^21^; the other was OCT4-ΔPE-GFP (hereafter mentioned as GFP), in which OCT4 PE element was deleted and OCT4 expression was primarily dependent on its distal enhancer activity as previousely reported^12^. Then, we genetically engineered primed embryonic stem cells (pESCs) with this reporter system and performed the primed-to-naive resetting under 5iLAF^14^ culture conditions (Fig. 1a). Small colonies were observed on day 6 of induction, when the expression of SSEA4 (primed state-specific surface marker) was dramatically decreased and a small portion of cells started to express SUSD2, a naive state-specific surface marker reported recently^28^ (Fig. 1b, c). Dome-shaped colonies resembling naive clones emerged on day 8, and the subpopulation of cells expressing RFP was greatly increased, with the proportion similar to the SUSD2^+^ cells (Fig. 1b, c). At this time, almost all the cells turned SSEA4-negative. GFP expression was not synchronized with RFP expression. The GFP^+^ subpopulation was not observable until day 10. The proportion of RFP^+^GFP^+^ cells increased along the induction process, and naive-like colonies were picked to establish naive ESC (nESC) lines on day 14 (Fig. 1b, c).

### Transcriptional profiling of intermediates during the primed-to-naive transition

Next, we collected the transitioning intermediates with distinct fluorescence dynamics through the primed-to-naive process, and subjected them to bulk RNA-seq (Supplementary Data 1). Principal component analysis (PCA) indicated that RFP^+^ cells collected since day 8 clustered closely with nESCs and were separated from pESCs (Fig. 1d, e). When integrated with human embryo datasets^29^, RFP^+^ cells and nESCs clustered closely with ICM cells, while the pESCs showed great similarities in transcriptome to reported blastocyst-derived hESCs at P0 and P10 (Supplementary Fig. 1a).

Toward naive pluripotency, epiblast-specific markers were upregulated starting on day 6 (Fig. 1f; Supplementary Fig. 1b), whereas primed state-specific genes were gradually downregulated (Supplementary Fig. 1b, Supplementary Data 2). Next, we characterized expression clusters based on the dynamics of gene expression (FPKM ≥ 5 in at least one sample) and identified six major expression patterns (Supplementary Fig. 1c–h, Supplementary Data 1). Primed pluripotency-associated genes participating in stem cell maintenance and embryonic morphogenesis were rapidly downregulated, including OTX2, ZIC2 and ZIC3 (Supplementary Fig. 1c), while naive pluripotency-related genes were activated in three categories: One group was enriched with genes essential for naive pluripotency regulation and mRNA processing, such as NANOG, TFAP2C, LIN28B and DPPA3, which showed increased expression from day 6 (Supplementary Fig. 1d); one group enriched with genes related to embryonic development and protein modification, such as DNMT3L and NODAL, showed peak expression on day 8 (Supplementary Fig. 1e); the third group was enriched with genes involved in oxidative phosphorylation metabolism, as well as core naive pluripotency markers ALPG and UTF1, the expression of which peaked on day 10 (Supplementary Fig. 1f). On day 6, SSEA4^−^ cells exhibited unique characteristics: one cluster with the transient upregulation of genes associated with PrE development (GATA4, GATA6), extracellular matrix organization (KRT8, COL1A1), and embryonic morphogenesis (HAND1, HAND2, HOXB4) (Supplementary Fig. 1g), and another cluster enriched with genes related to shared pluripotency markers, including POU5F1, SOX2, SALL4, etc., showing transient waves of downregulated expression on day 6 (Supplementary Fig. 1h). Thus, the bulk RNA-seq analysis uncovered different gene expression patterns during the primed-to-naive transition process.

### Charaterization of cell populations during the primed-to-naive transition at single-cell resolution

**Fig. 2:**
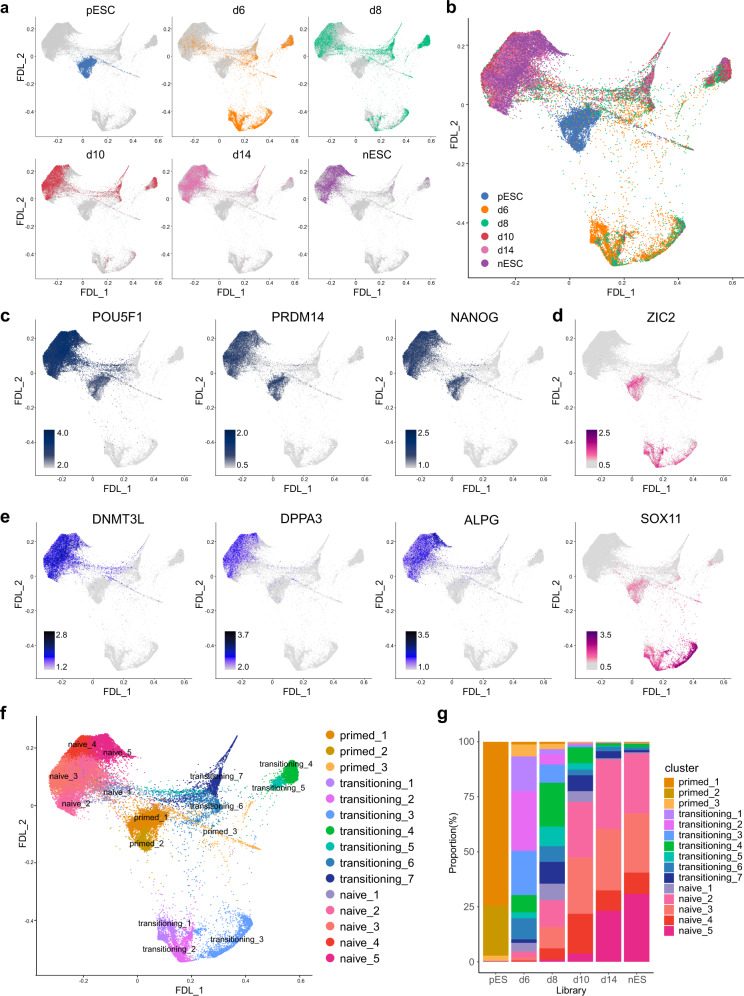
Single-cell transcriptome profiling during the primed-to-naive transition. **a** FDL highlighting cells within each time point or library. **b** FDL of the integrated scRNA-seq datasets (a total of 38,036 cells) with different libraries highlighted. **c**–**e** Expression of marker genes associated with shared pluripotency, POU5F1, PRDM14, NANOG (**c**, gray-blue); primed pluripotency,ZIC2, SOX11 (**d**, pink); naive pluripotency, DNMT3L, DPPA3, ALPG (**e**, blue) on FDL. **f** Cell clustering projection on FDL dimensionality reduction, total 15 clusters. **g** Bar plot showing cell clusters proportions of different libraries in (**f**). Source data are provided as a Source Data file.

RFP^+^ cells on day 8 showed similarities in transcription with RFP^+^GFP^−^ or RFP^+^GFP^+^ cells on days 10, 12, and 14, as well as nESCs (Fig. 1d, e; Supplementary Fig. 1a). Multiple naive pluripotency-related genes, such as ALPG, DNMT3L, DPPA3, KLF17 and SUSD2, showed robust upregulation during the primed-to-naive transition, and their expression levels reached a state comparable to that of naive pluripotency from day 8 (Fig. 1f). To further characterize the cell populations during the transition at single-cell resolution, we subjected cells harvested on days 6, 8, 10, and 14 during the transition process as well as human nESCs and pESCs to droplet-based 10× Genomics scRNA-seq, which generated a dataset of 38,036 cells with 16,929 common genes. The force-directed layout (FDL)^30^ shows the transitioning trajectory of intermediates and the relationships between single cells during the primed-to-naive transition (Fig. 2a, b). We also confirmed these findings by utilizing multiple dimensionality reduction methods to visualize cell embeddings in a low-dimensional space such as uniform manifold approximation and projection (UMAP) (Supplementary Fig. 2a–b), and t-distributed stochastic neighbor embedding (tSNE) (Supplementary Fig. 2c–d). Together with the expression patterns of known marker genes for shared pluripotency (POU5F1, PRDM14, NANOG, LEFTY1, TDGF1), primed pluripotency (ZIC2, SOX11) and naive pluripotency (DNMT3L, DPPA3, ALPG, DPPA5, FGF4) (Fig. 2c–e, Supplementary Fig. 3a–b), we identified and characterized 15 clusters during the transition process by performing unsupervised clustering analysis (Fig. 2f, Supplementary Fig. 3c–d). According to the cell proportions in different libraries or clusters, we observed that the populations with naive pluripotency signatures were greatly increased from day 8 (Fig. 2g, Supplementary Fig. 3e). Moreover, the subpopulations with ALPG expression emerging on day 8, also showed robust expression of other naive markers, such as DPPA3, DPPA5, and FGF4 (Supplementary Fig. 3f), which is consistent with our bulk RNA-seq analysis results.

### Chromatin accessibility dynamics during the primed-to-naive transition

Next, we tried to illustrate the chromatin accessibility landscape during the primed-to-naive transition process by transposase-accessible chromatin sequencing (ATAC-seq) of the intermediates according to their fluorescence dynamics (Supplementary Data 3–4). Conversion of OCT4 enhancer activity from the PE to the distal enhancer was observed during the primed-to-naive transition process (Supplementary Fig. 4a). Analyses of the repeatability among replicates, peak enrichment regions, and peak size distributions indicated the acquisition of ATAC-seq datasets with high-quality (Supplementary Fig. 4b–d).

**Fig. 3:**
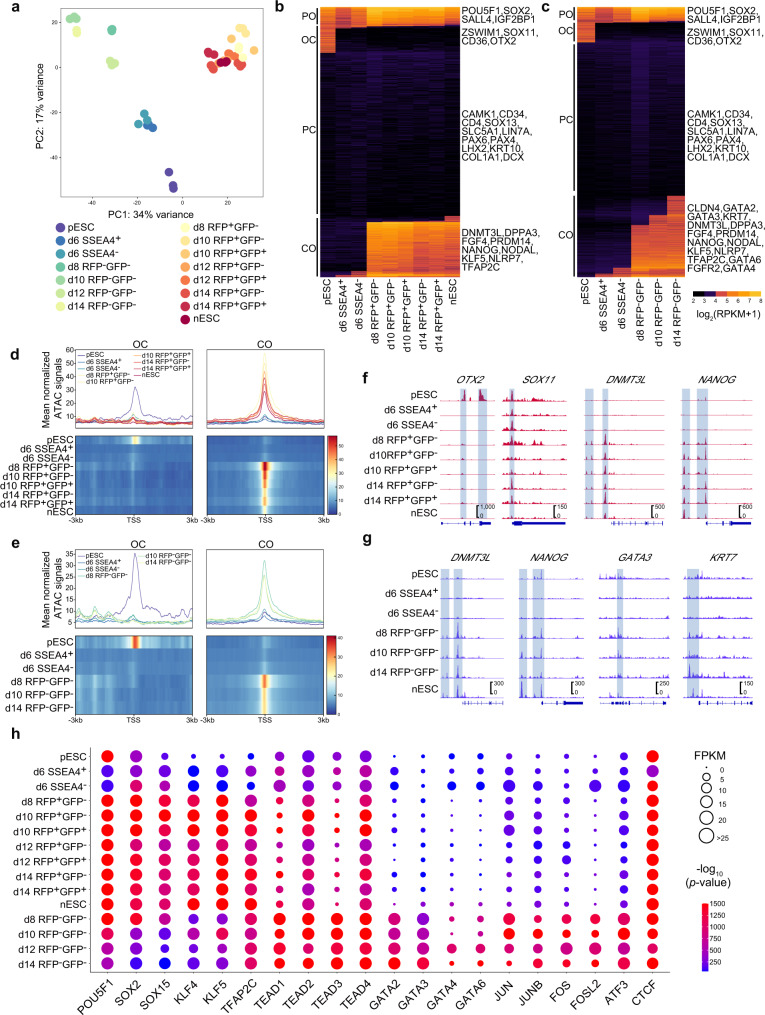
Chromatin accessibility dynamics during the primed-to-naive transition. **a** PCA of ATAC-seq datasets of the intermediate cells collected at different time points during the primed-to-naive transition process. *n* ≥ 2. **b** Chromatin loci arranged into groups according to closed or open status during the putative consecutive stages toward naive pluripotency. Representative genes are noted for each subgroup on the right side. CO closed to open, OC open-to-closed, PO permanently open, and PC permanently closed. **c** Chromatin loci arranged into groups according to closed or open status within the cells remaining RFP-negative. Representative genes are noted for each subgroup on the right side. CO closed to open, OC open-to-closed, PO permanently open, and PC permanently closed. **d**–**e** Profiles and heatmaps of ATAC signal on gene promoter regions (TSS ± 3 kb) within OC and CO groups in Fig. 2b (**d**) and Fig. 2c (**e**). **f**–**g** Representative ATAC-seq tracks for the OC and CO peaks of intermediates during the primed-to-naive transition. *OTX2* and *SOX11*, primed state-specific markers; *DNMT3L* and *NANOG*, naive state-specific markers; *GATA3* and *KRT7*, trophectoderm (TE)-specific markers. **h** Motif enrichment analysis of TFs. Colors and sizes represent motif enrichment (−log (*p* value)) and expression values (FPKM), respectively (*n* = 3 biologically independent samples). Source data are provided as a Source Data file.

PCA showed that all RFP^+^ cells correlated well with nESCs at different time points (Fig. 3a), indicating a similar chromatin accessibility state among cells with ALPG activation from day 8. Interestingly, cells that remained in the RFP^−^ state clustered closely, apart from the RFP^+^ cells (Fig. 3a; Supplementary Fig. 4b), suggesting cells with and without ALPG activation may have distinct chromatin landscapes and cell fates. Then we focused on the chromatin landscape differences between the two conditions: cells turning RFP-positive and cells remaining RFP-negative during the primed-to-naïve transition process. However, CAD charting revealed pattern similarities between the two conditions. (Fig. 3b–e; Supplementary Fig. 4e): The loci of genes involved in DNA repair, protein stability and in utero embryonic development were permanently open (PO) in both conditions, including those of the shared pluripotency factors *POU5F1* and *SOX2* (Fig. 3b, c; Supplementary Fig. 4e–f); the loci of differentiation-associated genes were permanently closed (PC), including those of *SOX13* and *PAX6* (Fig. 3b, c); the loci of genes involved in mRNA metabolic process and growth factor response underwent an open-to-closed transition (OC) in both two conditions, including primed state-specific factors *SOX11* and *OTX2* (Fig. 3b–f; Supplementary Fig. 4e, f). Different from the PO or OC gene loci that showed extremely high coincidence in both conditions, the loci that underwent a closed-to-open (CO) transition showed differences between the two conditions to some extend (Supplementary Fig. 4e). The loci of naive pluripotency-related genes, such as *DNMT3L* and *NANOG*, were opened not only in cells moving toward naive pluripotency but also in intermediates that remained RFP-negative (Fig. 3b–g), suggesting that these RFP^-^ cells may still possess the potential to reach the naive pluripotent state. Furthermore, the loci of both TE markers (*GATA3*, *KRT7* etc.) and PrE markers (*GATA4*, *GATA6*, *FGFR2*, etc.) are specifically in the CO category of CAD for intermediates remaining RFP-negative (Fig. 3c, e, g; Supplementary Fig. 4g). GO analysis of the genes within the CO categories showed that these genes are involved in embryonic epithelial tube formation and stem cell population maintenance in CADs composed of cells moving toward naive pluripotency and cells remaining RFP-negative, respectively (Supplementary Fig. 4f).

We also performed motif enrichment analysis among the ATAC-seq datasets. During the primed-to-naive transition process, the motifs of naive TFs (POU5F1-SOX2-TCF-NANOG, SOX2/15, and KLF4/5) were significantly enriched in RFP^+^ cells (Fig. 3h). The motifs of TE-specific TFs (TEAD family and GATA2/3) and TFs highly expressed in TSCs (JUN, JUNB, ATF3, FOS and FOSL2^31^) were enriched in RFP^−^ cells, which coincided with their corresponding TF expression (Fig. 3h). The TFAP2C motif was enriched in both RFP^+^ and RFP^−^ cells from day 8 (Fig. 3h), consistent with the activation of TFAP2C gene in both naive PSCs and TSCs. Thus, the analyses of the ATAC-seq datasets revealed clear TE signatures in RFP^−^ cells during the primed-to-naive transition process.

### TE signatures during the primed-to-naive transition

**Fig. 4:**
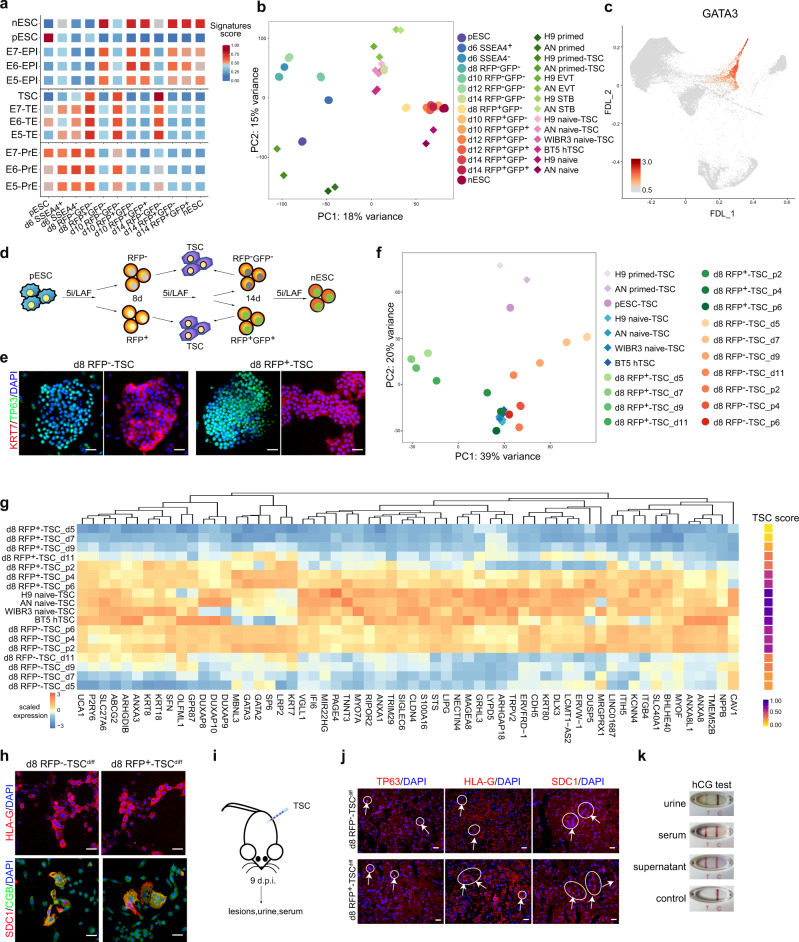
TE signatures during the primed-to-naive transition. **a** EPI, TE, PrE and trophoblast stem cell (TSC) signature scores of the primed-to-naive transitioning intermediates. **b** PCA of the bulk RNA-seq datasets (circles) from the primed-to-naive transitioning intermediates with published RNA-seq (diamonds) datasets^32^. *n* ≥ 2. **c** Expression of GATA3 in FDL. **d** Experimental design for the induction of TSCs from the primed-to-naive intermediates. **e** TP63 and KRT7 immunostaining of TSCs derived from day 8 RFP^−^ and day 8 RFP^+^ cells during the primed-to-naive transition. Scale bars, 20 μm. Representative images from *n* = 3. **f** PCA of the bulk RNA-seq datasets (circles) from the transitioning intermediates-derived TSCs with published RNA-seq (diamonds) datasets^32^. *n* ≥ 2. **g** Heatmap (left) and TSC score (right) showing the expression levels of representative TSC-related genes during the TSC derivation process from RFP^−^ and RFP^+^ transitioning intermediates on day 8. Source data are provided as a Source Data file. **h** HLA-G, SDC1 and CGB immunostaining of extravillous trophoblast (EVT) (upper) and syncytiotrophoblast (ST) (lower) cells, respectively. EVT and ST cells were differentiated from day 8-RFP^−^ and day 8-RFP^+^ cell-derived TSCs. Scale bar, 20 μm. Representative images from *n* = 3. **i** Representation of day 8-RFP^−^ and day 8 RFP^+^ cell-derived TSC engraftment assay by injection into NOD-SCID mice. **j** Immunostaining of TP63, HLA-G and SDC1 in the lesions collected from day 8-RFP^−^ and day 8 RFP^+^ cell-derived TSC engrafts in NOD-SCID mice. No lesions were evident in the vehicle controls. Scale bar, 20 μm. Representative images from *n* = 3. **k** Representative positive results for the hCG pregnancy test performed on urine samples, serum samples, and ST cell culture supernatant collected from day 8-RFP^−^ cell -derived TSCs. Source data are provided as a Source Data file.

We further characterized the TE signatures of RFP^−^ intermediates by RNA-seq analysis. Scoring analyses were performed to examine different embryonic signatures of the intermediate cells along the transition process (Fig. 4a; Supplementary Data 2). As expected, during the primed-to-naïve transition, signatures for the naive state and EPI were upregulated and maintained in RFP^+^ cells, while signatures for primed state was only enriched in pESCs (Fig. 4a). We also observed great enrichment of TE/TSC signatures in the RFP^−^ intermediates (Fig. 4a). In addition, when our bulk RNA-seq datasets were integrated with published TSC datasets^32^, RFP^−^GFP^−^ cells on day 14 clustered closely with TSCs derived from nESCs or blastocysts (Fig. 4b; Supplementary Fig. 5a). Representative TE markers, such as GATA2/3, KRT7 and TP63, were specifically expressed in RFP^−^ intermediates (Supplementary Fig. 5b), confirming their similarities with TE/TSCs in transcriptional programs. scRNA-seq analysis also showed that a subpopulation of RFP^-^ cells specifically expressed TE-associated genes (Fig. 4c; Supplementary Fig. 5c). Thus, together with the results of ATAC-seq analyses, we confirmed the appearance of a TE-like subpopulation with TE signatures within RFP^−^ intermediates.

### Derivation of TSCs from transitioning intermediate cells

Next, we speculated that this TE-like subpopulation could give rise to TSCs in vitro. We collected RFP^−^ intermediates on days 8 and 14 by flow cytometry and cultured them in TSC medium as previously reported^33^ (Fig. 4d). As TSCs can be generated from naive PSCs^7,32^, intermediate RFP^+^ cells were also subjected to TSC induction as controls. As expected, both RFP^−^ and RFP^+^ cells on day 8 or 14 successfully generated TSCs with representative colony morphologies and high expression of TSC markers, such as TP63 and KRT7 (Fig. 4e; Supplementary Fig. 5d–e), while pESCs failed to establish stable TSC lines, consistent with the results of previous studies^32^ (Supplementary Fig. 5f). Transcriptional profiling revealed high similarities among TSCs derived from RFP^−^ and RFP^+^ intermediates on day 8 of the primed-to-naive resetting, as well as TSCs described in published reports^32^ (Fig. 4f; Supplementary Data 5), further suggesting the capacity of these transitioning intermediates to generate TSCs. Interestingly, RFP^−^ cells and RFP^+^ cells may adopt different routes to establish TSCs during the TSC induction process, both with activation of TSC-related gene expression programs eventually (Fig. 4f, g). In addition, we also observed that RFP^-^ intermediates show accelerated trophoblast induction at early time points compared to RFP^+^ cells (Supplementary Fig. 5g).

We further assessed the differentiation potential of the TSCs derived from intermediates on day 8. These cells can differentiate into extravillous trophoblast (EVT) cells with specific HLA-G expression and syncytiotrophoblast (ST) cells with SDC1 and CGB expression when cultured under specific conditions^33^ (Fig. 4h). When subcutaneously injected into NOD-SCID mice, these TSCs generate lesions, as indicated by TP63^+^ cells, SDC1^+^ ST-like cells and HLA-G^+^ EVT-like cells observed by immunostaining (Fig. 4i, j). Moreover, human chorionic gonadotropin (hCG) was detected in the supernatant of ST cell cultures, urine and serum of the host mice injected with TSCs, as determined by pregnancy test sticks (Fig. 4k; Supplementary Fig. 5h). Taken together, the findings indicate that TSCs with functional differentiation capacities can be established from RFP^−^ cells, which exhibit TE signatures during the primed-to-naive transition.

### PrE signatures during the primed-to-naive transition

**Fig. 5:**
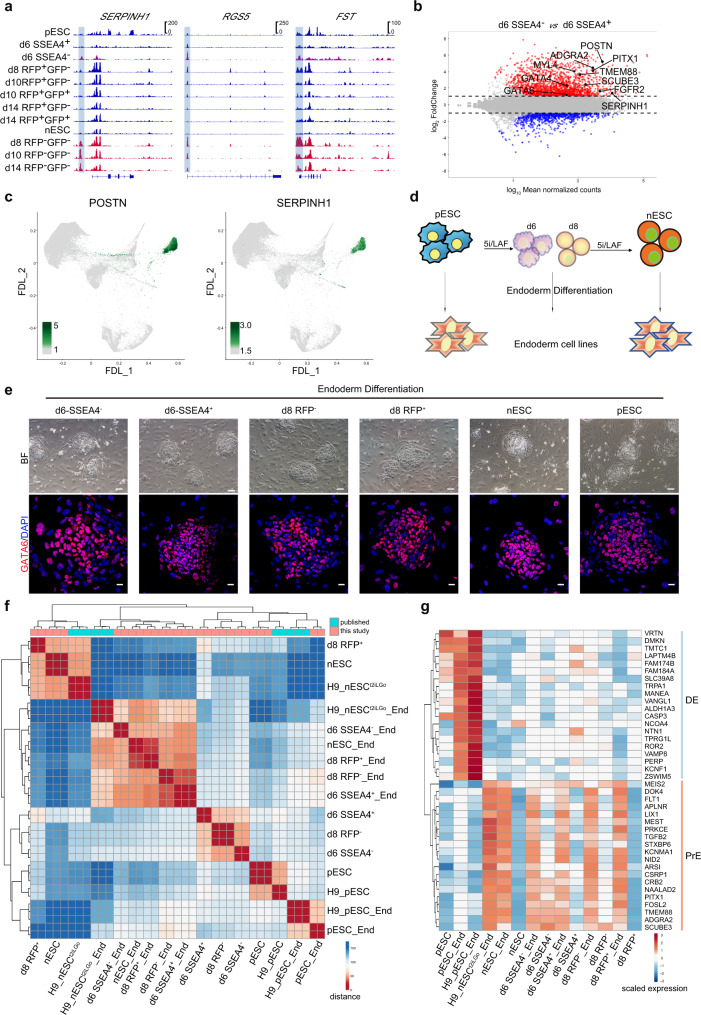
PrE signatures during the primed-to-naive transition. **a** ATAC-seq tracks showing the chromatin landscape of representative PrE-related genes in intermediate cells during the primed-to-naive transition. **b** MAplot of bulk RNA-seq datasets for comparing SSEA4^−^ cells with SSEA4^+^ cells on day 6 during the primed-to-naive transition with the differentially expressed (DE) genes highlighted. |log2FC | ≥ 1, adjusted *p* value < 0.01 (*n* = 3 biologically independent samples). **c** Expression of POSTN and SERPINH1 (marker gene of the PrE layer) as determined by FDL. **d** Experimental design for the induction of embryonic endoderm cell lines from the primed-to-naive intermediates following the reported protocol^38^. **e** Representative images (upper panels) and immunostaining (lower panels) showing the morphologies and GATA6 expression of endoderm cell lines derived from day6-SSEA4^−^ cells, day 6-SSEA4^+^ cells, day8 RFP- cells, day8 RFP^+^ cells, pESCs and nESCs. Scale bar (upper panels 50 μm, lower panels 20 μm). Representative images from *n* = 3. **f** Heatmap to indicate the samples distance among bulk RNA-seq datasets of endoderm cell lines derived from day6-SSEA4^−^ cells, day 6-SSEA4^+^ cells, day8 RFP- cells, day8 RFP^+^ cells, pESCs and nESCs with published RNA-seq datasets^34^, *n* ≥ 2. **g** Heatmap showing the expression levels of representative DE and PrE-related genes of endoderm cell lines derived from day6-SSEA4^−^ cells, day 6-SSEA4^+^ cells, day8 RFP- cells, day8 RFP^+^ cells, pESCs, nESCs and published endoderm cells^34^. Source data are provided as a Source Data file.

We also observed the appearance of PrE signatures during the primed-to-naïve transition process (Fig. 3h; Fig. 4a; Supplementary Fig. 1g). Motif enrichment analysis among the ATAC-seq datasets showed the motifs enrichment of endoderm markers GATA4 and GATA6 in RFP^−^ cells (Fig. 3h). Compared to RFP^+^ intermediates, ATAC-seq signals on PrE-related gene loci were more enriched in RFP^−^ and day 6-SSEA4^−^ intermediate cells (Fig. 5a). We also observed great enrichment of PrE signatures in both day 6-SSEA4^−^ intermediates and day 8-RFP^−^ cells by scoring analyses (Fig. 4a). Collectively, these data further indicated the appearance of PrE signatures.

Next, we tried to identify the PrE-like subpopulations during the transitioning process. As we detailed above, RNA-seq analysis revealed transient activation of PrE-associated markers, as well as the deactivation of core pluripotency markers, in the transitioning intermediates on day 6 (Supplementary Fig. 1g–h, Supplementary Data 1). Differential expression analysis confirmed the rapid upregulation of PrE-associated genes upon loss of SSEA4 expression (Fig. 5b; Supplementary Fig. 6a). The identity of SSEA4^−^ cell subpopulations on day 6, characterized by the 30 most differentially expressed (DE) genes, such as POSTN and PITX1, could also be determined by the expression of a series of PrE markers, including SERPINH1, FGFR2, MYL4 and TMEM88 (Fig. 5b, c; Supplementary Fig. 6b).

We then subjected the intermediates on day 6 and 8 of the transition process, as well as nESCs and pESCs, to endoderm differentiation respectively^34^ (Fig. 5d). All the derived endoderm cells could be maintained and expanded as stable cell lines with typical morphologies of embryonic endoderm cells and strong expression of endoderm marker gene GATA6 (Fig. 5e). However, these cell lines represent different characteristics corresponding to different embryonic development stages as reported^34^ (Fig. 5f).The endoderm cells derived from pESC (named as pESC_End cells) showed the definitive endoderm (DE) signatures with high expression of a series of DE genes (Fig. 5g, Supplementary Fig. 6c), while the endoderm cell lines derived from SSEA4^+^ and SSEA4^−^ cells on day 6, and RFP^−^ cells on day 8 of the primed-to-naive transition process, as well as nESC exhibit strong PrE signatures, with enriched expression of PrE-related genes (Fig. 5g, Supplementary Fig. 6c). Thus, the transitioning intermediates of the primed-to-naive transition, as well as nESCs, possess the potential to differentiate into endoderm cells with PrE signatures.

### Prolonged induction of RFP^-^ intermediates transitioning toward naive pluripotency

**Fig. 6:**
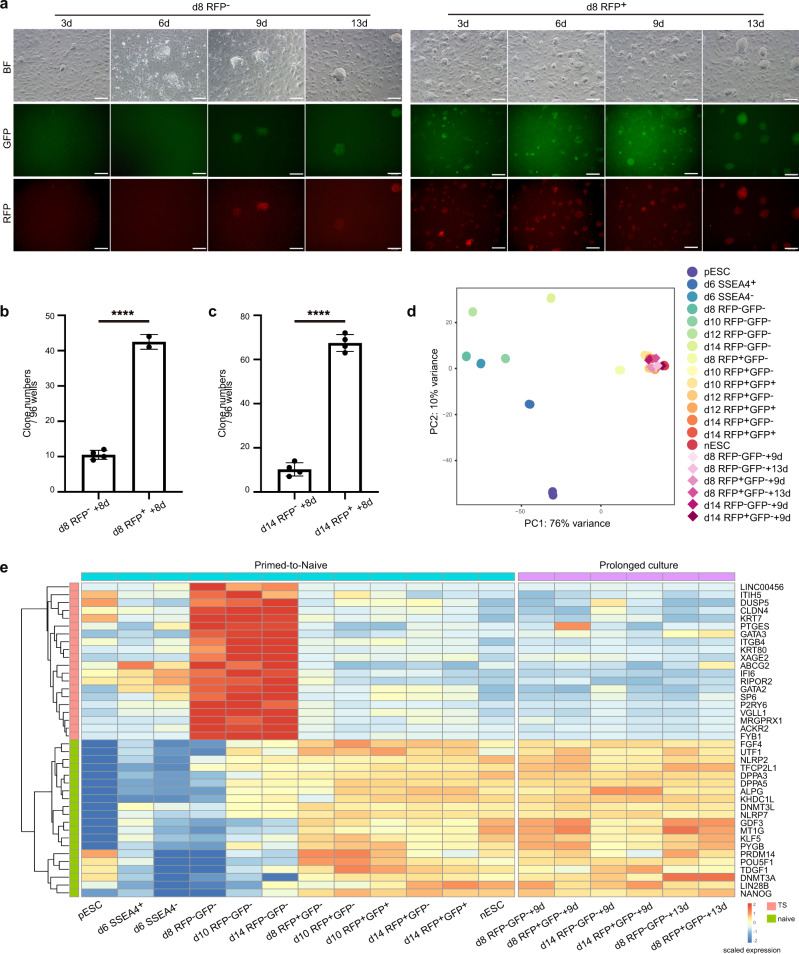
Prolonged induction of the RFP- intermediates transitioning toward naive pluripotency. **a** Morphological changes and fluorescent dynamics during prolonged 5iLAF culture of day 8-RFP^−^ cells (left) and day 8-RFP^+^ cells (right) from the primed-to-naive transition. Scale bar, 100 μm. Representative images from *n* = 5. **b** Statistical analysis of RFP^+^GFP^+^ colony numbers during prolonged 5iLAF culture of day 8-RFP^−^ cells and day 8-RFP^+^ cells from the primed-to-naive transition. 96-well plates (one cell/well) were counted (*n* = 4 and *n* = 2 biologically independent experiments respectively). ****p < 0.0001 (*p* = 2.5e^−07^), two-tailed Student’s *t* test. The error bars indicate the SD. Source data are provided as a Source Data file. **c** Statistical analysis of RFP^+^GFP^+^ colony numbers during prolonged 5iLAF culture of day 14-RFP^−^ cells and day 14-RFP^+^ cells from the primed-to-naive transition. 96-well plates (one cell/well) were counted (*n* = 4 biologically independent experiments). ****p < 0.0001 (*p* = 7.9e^−07^), two-tailed Student’s *t* test. The error bars indicate the SD. Source data are provided as a Source Data file. **d** PCA analysis of the bulk RNA-seq datasets (diamonds) from prolonged 5iLAF culture with the primed-to-naive transition intermediates (circles) datasets. *n* ≥ 2. **e** Expression dynamics of representative naïve pluripotency-related genes and TSC specific genes in subpopulations during the primed-to-naïve transition process. Source data are provided as a Source Data file.

As we observed a tendency of intermediate cells transitioning toward naive pluripotency by FDL (Fig. 2a), as well as the gradual opening of naïve state-related gene loci in RFP^−^ intermediates by CAD charting (Fig. 3c), we wondered whether the RFP^−^ intermediates can also reach naive state similar to the RFP^+^ cells by prolonged induction in 5iLAF medium. Although day 8-RFP^-^ cells remained fluorescence-negative for the first 6 days of prolonged 5iLAF culture, most cells became RFP^+^GFP^+^ after 9 days of prolonged induction in 5iLAF medium (Fig. 6a). Meanwhile, day 8-RFP^+^ cells converted into RFP^+^GFP^+^ cells more quickly than day 8-RFP^−^ cells (Fig. 6a). These results were also validated by subcloning assays, which showed higher efficiency of naive colony formation in day 8-RFP^+^ cells than day 8-RFP^−^ cells after 8 days of prolonged 5iLAF culture (Fig. 6b). Similar results could also be observed in prolonged 5iLAF culture of RFP^+^ and RFP^−^ cells on day 14 (Fig. 6c). These RFP^−^ intermediates finally reached a state resembling nESCs, as indicated by their transcription profiling, after prolonged induction in 5iLAF medium (Fig. 6d, e; Supplementary Data 5). Collectively, these results demonstrate the naive state-induction potential and developmental plasticity of RFP^−^ intermediates in the primed-to-naive transition.

### Cell fate roadmap of the primed-to-naive process

**Fig. 7:**
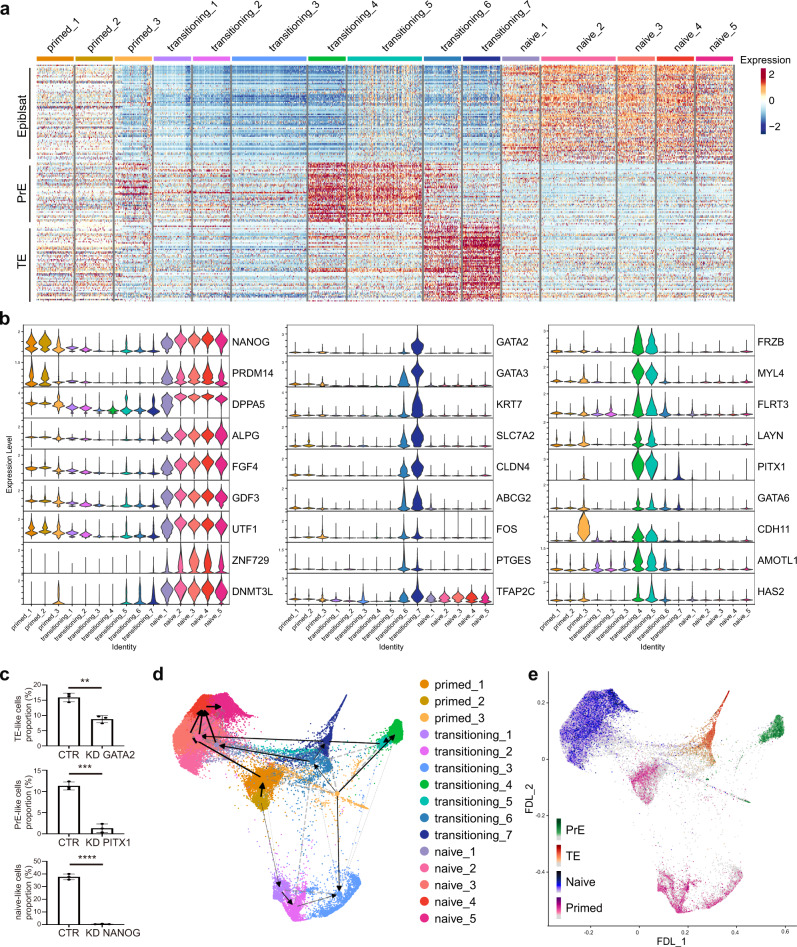
Cell fate roadmap of the primed-to-naive transition process. **a** Heatmap showing EPI, PrE and TE signatures in different cell clusters during the primed-to-naive transition. **b** Violin plots of representative naïve state-, TE-, and PrE-related genes in the 15 clusters classified in (**a**). **c** Statistical comparison of TE-, PrE- and naive-like cells proportions before and after knockdown of key branching-dependent transcription factors identified in (**b**) (*n* = 3 biologically independent experiments). **p < 0.01 (*p* = 0.0026), ***p < 0.001 (*p* = 0.0002), ****p < 0.0001 (*p* = 8.7e^−06^), two-tailed Student’s *t* test. CTR: control group, KD knockdown group. The error bars indicate the SD. Source data are provided as a Source Data file. **d** Directed PAGA-Velocity graph with PAGA connectivities (dashed) and transitions (solid/arrows) in a FDL. **e** Strength of naive (ALPG), primed (ZIC2), TE (GATA3) and PrE (SERPINH1) signatures in FDL.

Finally, we tried to describe the global cell fate trajectories of the primed-to-naive transition process. Together with the results above, We characterized and identified transitioning subpopulations with PrE signatures (transitioning subpopulations 4 and 5), TE signatures (transitioning subpopulations 6 and 7), and naive signatures according to the corresponding gene signatures and marker genes expression (Fig. 7a; Supplementary Fig. 7a), such as PITX1 and GATA6 for PrE signatures, GATA2 and GATA3 for TE signatures, and NANOG and ALPG for naive signatures (Fig. 7b). Moreover, knockdown of some of these branching-dependent transcription factors dramatically reduced the proportion of corresponding subpopulations, compared to the control group (Fig. 7c).

We also performed partition-based graph abstraction (PAGA)^35^ trajectory inference (Fig. 7d, Supplementary Fig. 7b–c) and RNA velocity^36,37^ computation (Fig. 7d, Supplementary Fig. 7d) to predict the future state of individual cells. Together with the latent time inference analysis (Supplementary Fig. 7e–f) that can reconstruct the temporal sequence of transcriptomic events, these results indicate that while a small proportion of cells at primed state can reach the naive state directly, most cells still undergo a more complicated transition: losing their primed pluripotency first, then appearing PrE and TE signatures successively in subpopulations, and acquiring the naïve pluripotency ultimately (Fig. 7e).

In conclusion, these results showed the cell fate roadmap from primed state toward naive state, in which subpopulations with PrE and TE signatures emerge successively, with a majority of cells ultimately reach the state with naive signatures.

## Discussion

In this study, we constructed a high-resolution roadmap to elucidate the cell fate transitions from the primed to naive pluripotency by a dual fluorescent reporter system via integration of transcription profiles and the chromatin accessibility landscape. Further investigation into transitioning cells with dynamic fluorescence indicated the appearance of PrE- and TE-like subpopulations with the capacity for extra-embryonic stem cell lines derivation including endoderm cell lines and TSC lines, respectively. However, the intermediate cells with either PrE or TE signatures could also acquire naive pluripotency by prolonged induction under naive conditions, strongly suggesting their developmental potential and diverse plasticity.

Using the dual fluorescent reporter system as well as SSEA4 antibodies, we collected cell populations according to their distinct fluorescence signals during the primed-to-naive process and identified several cell fate transitions by transcriptional analysis and ATAC-seq analysis. However, we also observed that a small proportion of day 6-intermediate cells had exhibited naïve signatures in advance despite the undetectable RFP^+^ signal in the scRNA-seq analysis results, suggesting that the first branching point of the cell fate trajectory might appear earlier. It is likely that there are epigenetic changes happening before and upstream of the observed transcriptional changes. Further studies invovled in epigenetic dynamics during the primed-to-naive transition can greatly deepen the understanding of the process.

Previous studies demonstrated the endoderm differentiation occurs in two waves during mammalian embryonic development. PrE in the preimplantation stage can predominantly give rise to extra-embryonic visceral yolk sac, and later some embryonic cells at gastrulation stage differentiate into definitive endoderm that contribute to embryonic organs. Naive PSCs are capable to differentiate into naïve extra-embryonic endoderm (nEnd) efficiently, which can be expanded as an in vitro study model for extra-embryonic PrE (hypoblast) development^34^. Here, we characterized subpopulations with PrE signatures during the primed-to-naïve transition, with the capacities to derive PrE-like endoderm cell lines. Different from endoderm stem cells differentiated from pESCs with definitive endoderm characteristics, these endoderm cells derived from the transitioning intermediates provide an in vitro culture model for extra-embryonic endoderm development studies.

During the past 5 years, diligent work and substantial advances have been made in human TSC derivation and culture condition optimization. Human TSCs can be derived from villous cytotrophoblast (CT) cells, blastocysts, and naive PSCs^7,31–33^, indicating powerful in vitro models that can be used to recapitulate human trophoblast development^31,33^. Excitingly, a recent study reported that human TSCs can even be induced directly from somatic cells, as indicated by a reprogramming roadmap analysis of transitioning somatic cells moving toward naive pluripotency^22^. In this study, we observed appearance of prominent TE-like and PrE-like subpopulations with corresponding signatures, which may mimic the postimplantation to preimplantation conversion in vitro. Moreover, we analyzed the dynamics of cell proportions with different lineage signatures during the primed-to-naive transition (Fig. 2g). The proportion of naive state-like cells was greatly upregulated on day 8 and ultimately reached 93% by the end of the transition process. The proportions of subpopulations with PrE or TE signatures increased from day 6 to day 8, then decreased sharply and were nearly undetectable by the end of the period.

Finally, the appearance of PrE-like, TE-like, and EPI-like (naive) cells on day 8 suggests the possibility that day 8-intermediates may serve as valuable sources for human blastocyst modeling and early embryogenesis studies.

## Methods

### Cell lines

293 T (human embryonic kidney cells) were acquired from ATCC (CRL-3216). The human primed ESCs with H9 background (kindly provided by Haoyi Wang, Institute of Zoology, CAS) were genetically engineered with ALPG-promoter-RFP (RFP) and OCT4-△PE-GFP (GFP) to generate the dual-fluorescence reporter cell lines. Human TSC and endoderm cell lines were generated from transitioning intermediates of the primed-to-naïve induction process. All research with human cell lines in this study complied with the principles laid out in the International Society for Stem Cell Research and with ethical approval for these experiments by the Biological Research Ethics Committee of Tongji University.

### Cell cultures

Human primed ESCs (pESCs) (H9) were cultured in conventional ESC medium containing DMEM/F12 (Thermo Fisher) with 20% KnockOut SR (Thermo Fisher), 1% nonessential amino acids (Millipore), 2 mM GlutaMAX (Millipore), penicillin-streptomycin (Millipore), and 8 ng/ml bFGF (PeproTech). The medium was changed daily, and the cells were passaged every 5 days using 0.5 mM EDTA (Invitrogen). Human nESCs derived from the corresponding pESCs were cultured in 5iLAF medium containing DMEM/F12:Neurobasal (1:1) (Thermo Fisher), 1% N2 supplement (Thermo Fisher), 2% B27 supplement (Thermo Fisher), 0.5% KnockOut SR (Thermo Fisher), 1% nonessential amino acids (Millipore), 2 mM GlutaMAX (Millipore), penicillin-streptomycin (Millipore), 20 ng/ml human LIF (Millipore), 8 ng/ml bFGF (PeproTech), 50 μg/ml BSA (Sigma) and the following cytokines and small molecules: 1 μM PD0325901 (Selleck), 0.5 μM SB590885 (Selleck), 1 μM WH-4-023 (Selleck), 10 μM Y-27632 (Selleck), and 20 ng/ml activin A (PeproTech), and passaged with Accutase (Sigma) every 4-5 days as previously reported^14^. Human TSCs were cultured in TSC medium containing DMEM/F-12 supplemented with 0.3% BSA (Sigma), 0.2% FBS (Thermo Fisher), a 1% ITS-X supplement (Thermo Fisher), 0.1 mM 2-mercaptoethanol (Millipore), 2 mM GlutaMAX (Millipore), penicillin-streptomycin (Millipore), 1.5 μg/ml l-ascorbic acid (Sigma), 5 μM Y27632 (Selleck), 2 μM CHIR99021 (Selleck), 0.5 μM A83-01 (Sigma), 1 μM SB431542 (Selleck), 50 ng/ml EGF (PeproTech) and 0.8 mM valproic acid (VPA) (Sigma). The medium was changed daily, and the cells were passaged every 2–4 days using 0.5 mM EDTA (Invitrogen). Human endoderm cells were derived and cultured in RACL for 6–7 days and then in NACL medium following the protocol described previously^38^. The RACL medium was prepared containing: RPMI 1640 medium (Thermo Fisher), GlutaMAX (Thermo Fisher), B27 minus insulin (Thermo Fisher), 100 ng/ml Activin A (PeproTech), 3 µM CHIR99021 (Selleck) and 10 ng/ml recombinant human LIF (PeproTech). The NACL medium was prepared containing: DMEM/F12: Neurobasal (1:1) (Thermo Fisher), 1% N2 supplement (Thermo Fisher), 1% B27 supplement (Thermo Fisher), 1% GlutaMAX (Millipore), 1%nonessential amino acids (Millipore), 0.1 mM β-mercaptoethanol (Sigma), 0.5% penicillin–streptomycin (Millipore), 100 ng/ml Activin A (PeproTech), 3 μM CHIR99021 (Selleck) and 10 ng/ml recombinant human LIF (PeproTech). Cells were passaged with Accutase (Sigma) every 4-7 days. All human cell lines were cultured in 5% O_2_ and 5% CO_2_ at 37 °C. Mycoplasma tests were performed every week. Human ESC lines were used in accordance with the ethical approvals obtained from the Biological Research Ethics Committee of Tongji University.

### The primed-to-naive transition

The generation of ALPG-promoter-RFP (RFP); OCT4-△PE-GFP (GFP) pESCs was performed as previously described^14,21^. In brief, the ALPG promoter was cloned into a pSicoR-RFP plasmid (Addgene) to replace the CMV promoter, and was transiently co-transfected with packaging plasmids into 293 T cells. After 48 h, the viral supernatants were harvested, concentrated and incubated with OCT4-△PE-GFP pESCs. For inducing the primed to naive state transition, 2–3 × 10^5^ dissociated single RFP; GFP pESCs were seeded on an irradiated feeder layer in conventional ESC medium supplemented with Y-27632 (Selleck, 10 mM). The medium was then switched to 5iLAF medium on the second day and was changed every day thereafter. The intermediate cells were identified by flow cytometry analysis and collected at different time points during the primed-to-naive transition.

### Bulk RNA-seq library generation and sequencing

Total RNA was isolated from cells using TRIzol (Invitrogen). To generate RNA-sequencing libraries, a KAPA stranded mRNA-Seq kit (KAPA) was used following the manufacturer’s instructions. Adapters were through a TruSeq Library Prep Pooling kit (Illumina). Paired-end150 bp sequencing was further performed on a Novaseq 6000 (Illumina) at Berry Genomics Corporation.

### RNA-seq data processing

RNA-seq raw reads were processed with default parameters by Trim_galore (version 0.6.6) to remove adapters and low-quality reads. Bulk RNA-seq reads were then aligned to the human genome (hg38) using STAR (STAR_2.5.2b)^39^ with the default parameters except for “--outSAMattrIHstart 0”, “--outSAMstrandField intronMotif”, “--outFilterIntronMotifs RemoveNoncanonical”, “--outFilterMismatchNmax 999”, “--outFilterMismatchNoverReadLmax 0.04”, “--quantMode GeneCounts”, and “--twopassMode Basic” parameters. Expression levels of all Refseq genes for samples were quantified to FPKM using Stringtie (version 2.1.4)^40^. To perform differential gene expression analysis, the RNA-seq raw counts calculated by STAR were processed by DEseq2^41^, and genes with a Benjamini-Hochberg adjusted *p* value < 0.05 and a fold change >2 were considered DE. For principal component analysis (PCA) of the RNA-seq data, the rlog() and plotPCA() functions with the “returnData=T” parameter in the DEseq2 package were used to normalize the counts and compute the PCA data. PCA data were then plotted with the ggplot2 package in R (http://ggplot2.org). Pearson correlation coefficient between samples was calculated using the R function cor(), and heatmap was plotted by the pheatmap package in R. K-means clustering was performed for genes with FPKM ≥ 5 in at least one sample among the selected stages setting k = 6. For public RNA-seq datasets, we downloaded the raw data and performed de novo analysis to obtain the raw counts and FPKM of the samples. To perform the integrated PCA with our all RNA-seq samples, we first merged the raw counts and performed normalization using the vst() function in the DEseq2 package, and the batch effects of the samples were removed using removeBatchEffect function in the limma package in R, then PCA was performed with all genes by the R prcomp()function. The sample distance was calculated by the R hclust() function by the “ward.D2” method.

### ATAC-seq library generation and sequencing

ATAC-seq was performed as previously described^42^. In brief, a total of 50,000 cells were washed once with 50 μl of cold PBS, centrifuged for 5 min at 500 g at 4 °C, resuspended in 50 μl of lysis buffer (10 mM Tris-HCl (pH 7.4), 10 mM NaCl, 3 mM MgCl_2_, and 0.1% (v/v) NP40 and incubated on ice for 10 min. The suspension with nuclei was then centrifuged for 5 min at 500 *g* at 4 °C, and 50 μl of a transposition reaction mixture (10 μl of 5× TTBL, 5 μl of TTE Mix V50 and 35 μl of nuclease-free H_2_O) obtained through a TruePrep DNA Library Prep Kit V2 for Illumina (TD501-TD503, Vazyme) was added, and the mixture was incubated at 37 °C for 30 min. DNA fragments were isolated using a MinElute kit (QIAGEN). ATAC-seq libraries were processed through 13 cycles of amplification with a TruePrep DNA Library Prep Kit V2 for Illumina (TD501-TD503, Vazyme) according to the manufacturer’s instructions, and then, the libraries were purified using a QIAquick PCR (QIAGEN) column. The library concentration was measured using Qubit kit according to the manufacturer’s instructions. Finally, the ATAC library was sequenced on Novaseq 6000 (Illumina) at Berry Genomics Corporation.

### ATAC-seq data processing

The ATAC-seq sequencing data were preprocessed with the default parameters by Trim_galore (version 0.6.6)^43^ to remove adapters and low-quality reads. All the cleaned reads were aligned to the human genome assembly (hg38) using bowtie2 (version 2.4.1)^44^ with the default parameters except for the following options: “-X 2000 --no-unal --very-sensitive”. Reads mapping to mitochondrial DNA were discarded using the “grep –v chrM” command. Only high-quality mapped reads and concordantly aligned pairs were retained using SAMtools (view –q 30 -f 2)^45^. For downstream analysis, PCA duplicates were removed using the sambamba markdup function (version 0.7.1)^46^ with “-r” parameters. Alignment BAM files were transformed into read coverage files (bigWig format) using deepTools (version 3.5.0)^47^ through the RPKM normalization method, and the hg38 blacklist regions were also removed using “--blackListFileName” parameters. Biological replicates with high correlation were merged, and peaks were called using MACS2 (version 2.2.7.1)^48^ with default options except for the following options: --nomode -f BAMPE --keep-dup all. A motif analysis was performed using HOMER (v.4.11.1)^49^ “findMotifsGenome.pl” function with the “-size given” option. For PCA analysis, ATAC-seq peaks across all samples were merged into one union ATAC-seq peak set using the BEDTools^50^ merge function, and ATAC-seq reads in each sample were calculated over the union ATAC-seq peak set using the deepTools multiBigwigSummary function with RPKM-normalizd bigWig files. The output matrix was then log2 transformed (log2 + 1) and used as input for the PCA. The variance of normalized ATAC-seq reads over each peak was then calculated, and PCA analysis (prcomp function in R) was performed on peaks with the highest 2000 variances across samples. PCA plots were then plotted with the ggplot2 package in R. The Pearson correlation coefficient was calculated for samples using the R function cor(), and a heatmap was plotted by the pheatmap package in R. Peak annotation of the union ATAC-seq peak set was performed by chIPseeker^51^. For the definition of the “open” or “closed” state of ATAC-seq peaks, the background regions in the genome were first randomly identified, and the combined ATAC-seq peak set regions were excluded using the BEDTools shuffle function with the “-excl” parameter. Then, the ATAC-seq reads in each sample were calculated over the background regions similarly to the aforementioned analysis. We calculated the false discovery rate (FDR) between the peak region matrix and the background region matrix, setting the peak threshold RPKM value to 14.22, which resulted in a 1% false discovery rate. All downstream analyses were based on this threshold value: Reads with a value below this threshold were annotated to indicate “closed” loci, while those with a value above the threshold were considered to be “opened” loci.

### Single-cell RNA-seq data processing and integration

The 10× Genomics single-cell data were preprocessed using the Cell Ranger pipeline (v.4.0.0) with default parameters to generate the expression matrix. For quality control, all cutoffs were determined after investigating the distributions of each variable. Cells with a low number of expressed genes (nFeature), extremely high counts (nCount) or a high percentage of mitochondrial genes (pctMT) were discarded. The following thresholds were applied to retain cells: nFeature >2500, 1000 <nCount <100,000 and pctMT < 10. Genes not present in at least 10 cells with at least 1 read each were discarded. Ribosomal genes were also removed from downstream analysis. After quality control, 5707 cells and 18,826 genes remained in pESC samples, 6812 cells and 21,069 genes remained in the day 6 samples, 7175 cells and 21,659 genes remained in the day 8 samples, and 7035 cells and 21,004 genes remained in the day 10 samples, and 6831 cells and 19,978 genes remained in the day 14 samples, and 4476 cells and 19,809 genes remained in nESC samples.To correct for technical differences and to perform an integrated analysis with our single-cell data, we utilized the Seurat v.3 integration technique (v.3.2.3)^52^ and followed official protocols provided by Satija Lab to integrate the different datasets. In brief, the functions NormalizeData (with default settings) and FindVariableFeatures (using 2000 features) were applied to the datasets separately, and then, the functions FindIntegrationAnchors (using 30 dimensions) and IntegrateData (using common genes) were applied to integrate the datasets. This resulted in an integrated single-cell dataset comprising 38,036 cells and 16,929 genes.

### Single-cell RNA-seq dimension reduction, trajectory inference and RNA velocity analysis

For the dimension reduction, PCA was performed on the scaled gene expression using the RunPCA function in Seurat package. Following that, UMAP and t-SNE were implemented on the top 24 PCs via the RunUMAP and RunTSNE functions, respectively. FDL was generated using the scanpy.tl.draw_graph function in the scanpy package using the ForceAtlas 2 layout and initialized using the UMAP coordinates.

Partition-based graph abstraction (PAGA)^35^ method was utilized to perform trajectory inference with preserving the global topology of data, which is robust and qualitatively outperforms previous lineage reconstruction algorithms. The PAGA algorithm was performed using the scanpy.tl.paga function in the scanpy package (v.1.7.2) using the Seurat cell clusters as input.

For RNA velocity analysis, spliced and unspliced matrices of reads were summarized using velocyto (v.0.17.17)^36^ with default parameters. scVelo^37^ was performed for RNA velocity analysis (v.0.2.4) The non-default scVelo parameters were: velocity_mode = ‘dynamical’, n_top_genes = 500.PAGA-velocity matrix were computed by the function scVelo.tl.paga and ploted by scVelo.pl.paga.

### Scoring of single-cell RNA-seq and bulk RNA-seq samples using primed or naive gene signatures and TE, EPI, DE, PrE and TSC signatures

Scores of the gene signatures (EPI, TE, and PE) of single-cell RNA-seq was calculated with the AddModuleScore function in Seurat. Scores of the different gene signatures in the bulk RNA-seq samples were calculated as previously described^22^. In brief, the expression range value (max - min) for each gene across all samples was first computed. Then, the scores of each gene of the gene set across all samples were computed by the formula: (gene expression − min)/(max − min), obtaining scaled gene expression ranging from 0 to 1. Finally, the sample score of the gene signatures was the mean expression of all the gene scores per sample. The primed, naive, TE, EPI, DE, and PE gene sets were obtained from the paper^53,54^, and the list of TSC marker genes was defined by the relative gene expression (FPKM) of nTSCs compared to human ESCs (naïve and primed), primed TSCs, EVT and ST cells. Genes with log2((nTSC + 1)/(hES + 1)) > 3, log2((nTSC + 1)/(pTSC + 1)) > 2.5, log2((EVT + 1)/(nTSC + 1)) < −1.5 and log2((ST + 1)/(nTSC + 1)) < −1.5 were kept as TSC markers, RNA-seq data of TSCs, EVT and ST were from this paper^32^. For the score computation in this study, we only retained the genes with FPKM ≥ 5 in at least 1 sample in the gene set. Descriptions of these gene sets can be found in Supplemental Data.

### Immunostaining and flow cytometry

For immunostaining, cells were fixed overnight with PBS (Thermo Fisher) containing 4% paraformaldehyde (Sigma) at 4 °C and permeabilized for 15 min in PBS containing 0.05% Triton X-100. After incubation with blocking buffer (PBS containing 4% BSA) for 30 min at room temperature, the cells were incubated with primary antibodies followed by secondary antibodies. The following primary antibodies used in this study were used: APC-conjugated anti human SSEA4 (BioLegend, Cat#330407,RRID: AB_1089201—1:50 dilution), APC-conjugated anti-KRT7 (Abcam, Cat#ab192077, Lot GR3214132-7,1:500 dilution), anti-TP63 (Cell Signaling, Cat#13109 T, Lot 3, 1:800 dilution), anti-HLA-G (Abcam, Cat#ab7759, RRID:AB_306053, Lot GR3262011-5, 1:500 dilution), anti-CGB (Abcam, Cat#ab131170, RRID:ab_11156864, 1:500 dilution), and anti-SDC1 (Abcam, Cat#ab181789, Lot GR317857, 1:500 dilution), Goat anti-GATA6 (R&D Systems,Cat#AF1700, Lot KWT0418111, 1:200 dilution), Anti-SUSD2 Mouse Monoclonal Antibody (APC) (BioLegend, Cat#327408, clone:W5C5, RRID:AB_2561888, 1:50 dilution). Alexa Fluor conjugated secondary antibodies were then used: 488 Donkey Anti-Rabbit IgG (H + L) Antibody (Invitrogen, Cat#A-21206, RRID:AB_2535792, 1:500 dilution), 488 Donkey Anti-Mouse IgG (H + L) Antibody (Thermo Scientific, Cat#A-21202, RRID:AB_2536180, 1:500 dilution), 594 Donkey Anti-Rabbit IgG (H + L) Antibody (Fisher Scientific, Cat#A-21207, RRID:AB_141637, 1:500 dilution), 594 Donkey Anti-Mouse IgG (H + L) Antibody (Fisher Scientific, Cat#A-21203, RRID:AB_141633, 1:500 dilution), 647 Donkey Anti-Rabbit IgG (H + L) Antibody (Thermo Scientific, Cat#A-31573, RRID:AB_2536183, 1:500 dilution), 47 Donkey Anti-Mouse IgG (H + L) Antibody (Thermo Scientific, Cat#A-31571, RRID:AB_162542, 1:500 dilution). Nuclei were stained with 4’,6-diamidino-2-phenylindole (Sigma, Cat#D8417, 1:1000 dilution). Images were taken using the Zeiss LSM880 microscope system. For flow cytometry, cells were collected and washed with FACS buffer containing PBS supplemented with 2% FBS. The cells were washed and resuspended in FACS buffer after staining with APC-conjugated anti-SSEA4 (BioLegend) or APC-conjugated anti-KRT7 (Abcam) antibodies. All analyses were performed on Cytoflex S (Beckman Coulter) and MoFlo Astrios^EQ^ cell sorter (Beckman Coulter). Flow cytometry data were processed using Flow Jo software (V10.0).

### Differentiation of TSCs and genes knockdown experiments

For differentiation of TSCs in vitro, TSCs cultured to 80% confluence were dissociated by TrypLE select (Thermo Fisher) and then seeded onto a 6-well plate precoated with 1 mg/ml Col IV (Sigma) at a density of 2 × 10^5^ cells per well. For induction of EVT cells, the cells were cultured in EVT medium containing DMEM/F12 supplemented with 0.1 mM 2-mercaptoethanol (Millipore), penicillin-streptomycin (Millipore), 0.3% BSA (Sigma), 1% ITS-X supplement (Thermo Fisher), 100 ng/ml NRG1 (Cell Signaling), 7.5 μM A83-01 (Selleck), 2.5 μM Y27632 (Selleck), and 4% KnockOut Serum Replacement (Thermo Fisher). Matrigel (Corning) was added to a final concentration of 2% shortly after suspending the cells in the medium. The medium was replaced with EVT medium without NRG1 (Cell Signaling), and Matrigel (Corning) was added to a final concentration of 0.5% after 3 days of induction. The cells were analyzed on day 5. For differentiation into ST cells, the cells were cultured in ST medium containing DMEM/F12 supplemented with 0.1 mM 2-mercaptoethanol (Millipore), penicillin-streptomycin (Millipore), 0.3% BSA (Sigma), 1% ITS-X supplement (Thermo Fisher), 2.5 μM Y27632 (Selleck), 2 μM forskolin (Selleck), and 4% Knockout SR (Millipore). The medium was replaced on day 3, and the cells were analyzed on day 6. For differentiation of TSCs in vivo, 10^7^ TSCs were resuspended in 200 μl of a 1:2 mixture of Matrigel (Corning) and DMEM/F-12 with GlutaMAX (Thermo Fisher) supplemented with 0.3% BSA (Sigma) and 1% ITS-X (Thermo Fisher) and then injected subcutaneously into the dorsal flanks of 5–20-week-old male and female NOD/SCID IL-2R-gamma-knockout mice (100 μl were injected into each flank). The specfic pathogen-free grade mice (SPF) grade mice were housed under a 12 h light/dark cycle under pathogenfree conditions at 22.1–22.3 °C and 33–44% humidity, and fed with free access to standard mouse chow and tap water in the animal facility at Tongji University, Shanghai, China. Nine days after injection, mouse urine and blood serum were assessed for the detection of hCG secretions. Lesions were fixed overnight with 4% PFA (Sigma) and subsequently embedded in paraffin, sectioned and subjected to immunostaining as described above. All experiments were performed in accordance with the University of Health Guide for the Care and Use of Laboratory Animals and were approved by the Biological Research Ethics Committee of Tongji University.

For knockdown experiments, shRNA specifically targeting GATA2, PITX1 and NANOG gene were designed and cloned into pSicoR vector (Addgene, 11579), which was transiently co-transfected with package plasmids into 293 T cells, respectively. After 48 h, viral supernatants were harvested, concentrated and incubated with primed cells. Then, we performed the primed-to-naive transition and collected intermediate cells on day6 for PITX1 knockdown experiment, and day8 for GATA2 and NANOG experiment. Cell subpopulations were validated by flow cytometry. GATA2 shRNA target sequences: CTACAAGCTGCACAATGTTAA, CCGGCACCTGTTGTGCAAATT; PITX1 shRNA target sequences: GCAACGTACGCACTTCACAAG, GCACTTCACAAGCCAGCAGTT; NANOG shRNA target sequences: GCATCCGACTGTAAAGAATCT, GCAAATGTCTTCTGCTGAGAT.

### Statistical analyses

For flow cytometry analysis and immunostaining, *n* = 3 biologically independent replicates were included for each sample. For bulk RNA-seq data of the intermediate cells during the primed-to-naive transition process, *n* = 2 biological replicates were obtained for each sample at each time point during the transition process, except for pESCs (*n* = 3), nESCs (*n* = 3) and RFP^-^GFP^−^ cells on day 8 (*n* = 4). For ATAC-seq, *n* = 3 biological replicates were obtained for each sample at each time point except for RFP^+^GFP^−^ cells on day 14 (*n* = 6). For 10× Genomics scRNA-seq data, libraries were generated on pESC (*n* = 1), day 6 (*n* = 1), day 8 (*n* = 1), day 10 (*n* = 1), day 14 (*n* = 1) and nESC (*n* = 1). The number of cells used for downstream analysis were 5707 for the pES library, 6812 for the day 6 library, 7175 for day 8 library, 7035 for day 10 library, 6831 for the day 14 library, 4476 for the nES library. For the bulk RNA-seq data of intermediate cells toward TSC induction and those in prolonged 5iLAF culture toward naive pluripotency respectively, *n* = 2 biological replicates were obtained for each sample. Detailed information can be found in specific parts of the Methods section.

### Reporting summary

Further information on research design is available in the Nature Research Reporting Summary linked to this article.

## Supplementary information

Supplementary Information

Description of Additional Supplementary Files

Supplementary Data 1

Supplementary Data 2

Supplementary Data 3

Supplementary Data 4

Supplementary Data 5

Reporting Summary

## Data Availability

The bulk RNA-seq datasets, scRNA-seq datasets and ATAC-seq datasets generated in this study are available at GEO: GSE173756 and GSE174771. The accession number for the RNA-seq data of human embryos is GSE36552. The accession numbers for the RNA-seq data of published TSC cell lines is GSE138762. The accession number for the RNA-seq data of End cell lines is GSE138012. Source data are provided with this paper.

## Source data

Source Data

## Code availability

All data were analyzed with standard programs and packages as detailed. Scripts can be found at https://github.com/zftu/Human-primed-to-naive-transition-analysis^55^.
