# Supplementary material for: Cell fate roadmap of human primed-to-naive transition reveals preimplantation cell lineage signatures
Source: Nat Commun. 2022 Jun 7;13:3147. doi: 10.1038/s41467-022-30924-1 (PMC9174176; doi:10.1038/s41467-022-30924-1)
Supplement: Supplementary file 1 — Supplementary Information [file 41467_2022_30924_MOESM1_ESM.pdf]

Supplementary Figures

Supplementary Fig. 1

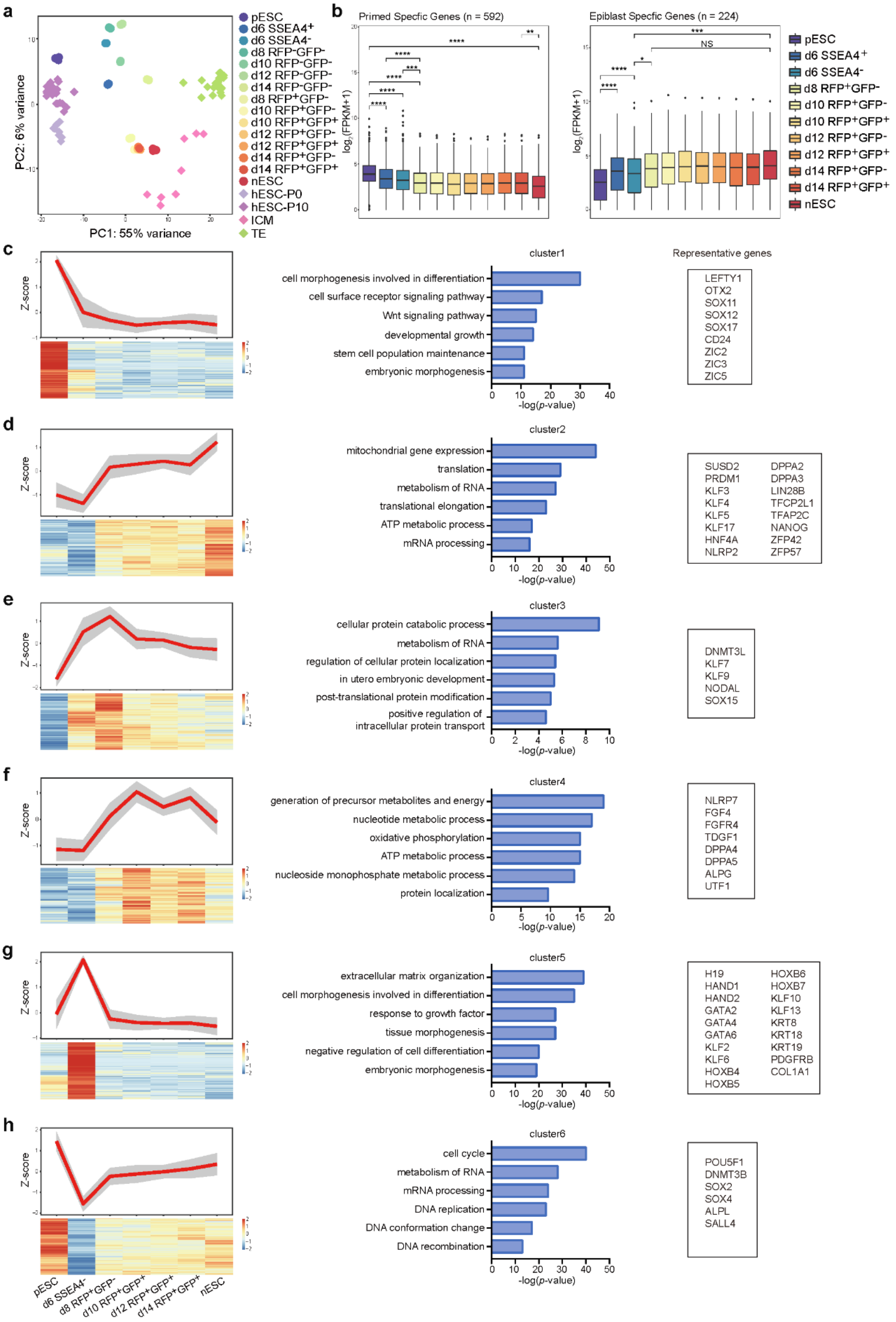

Supplementary Fig. 1 | Bulk RNA-seq analysis of expression dynamics during the primed-to-naive transition process.

a, PCA of the bulk RNA-seq datasets of the primed-to-naive transitioning intermediates collected at different time points with the published datasets[29]. $n \geqslant 2$.

b, Boxplots showing the expression of primed state-specific genes (left panel) and epiblast state-specific genes (right panel) during the primed-to-naive transition process (n=2 biologically independent samples). $*p < 0.05$, $**p < 0.01$, $***p < 0.001$, $****p < 0.0001$, two-tailed Student's t test. NS, no significant. Right panel: p-values from top to bottom are (0.00072, 0.28, 0.024, $2.6e^{-06}$, $3.5e^{-08}$); Left panel: p-values from top to bottom are (0.0022, $2.22e^{-16}$, $3.2e^{-06}$, 0.00021, $2.22e^{-16}$, $2.22e^{-16}$, $4e^{-13}$, $1.5e^{-11}$). The middle lines in the boxes indicate the medians. The box hinges indicate the 25th and 75th percentiles, and the whiskers indicate the hinge ± 1.5 interquartile ranges.

c-h, Line plots and heatmaps (left panels) showing the expression patterns of genes in cluster 1 (c), cluster 2 (d), cluster 3 (e), cluster 4 (f), cluster 5 (g), and cluster 6 (h). Solid lines and ribbons of the line plots represent mean of scaled gene expression across clusters ± s.d; Bar plot (middle panels) showing enrichment of *p*-value of the representative gene ontology (GO) term of each cluster, with representative genes listed in the right panels.

Supplementary Fig. 2

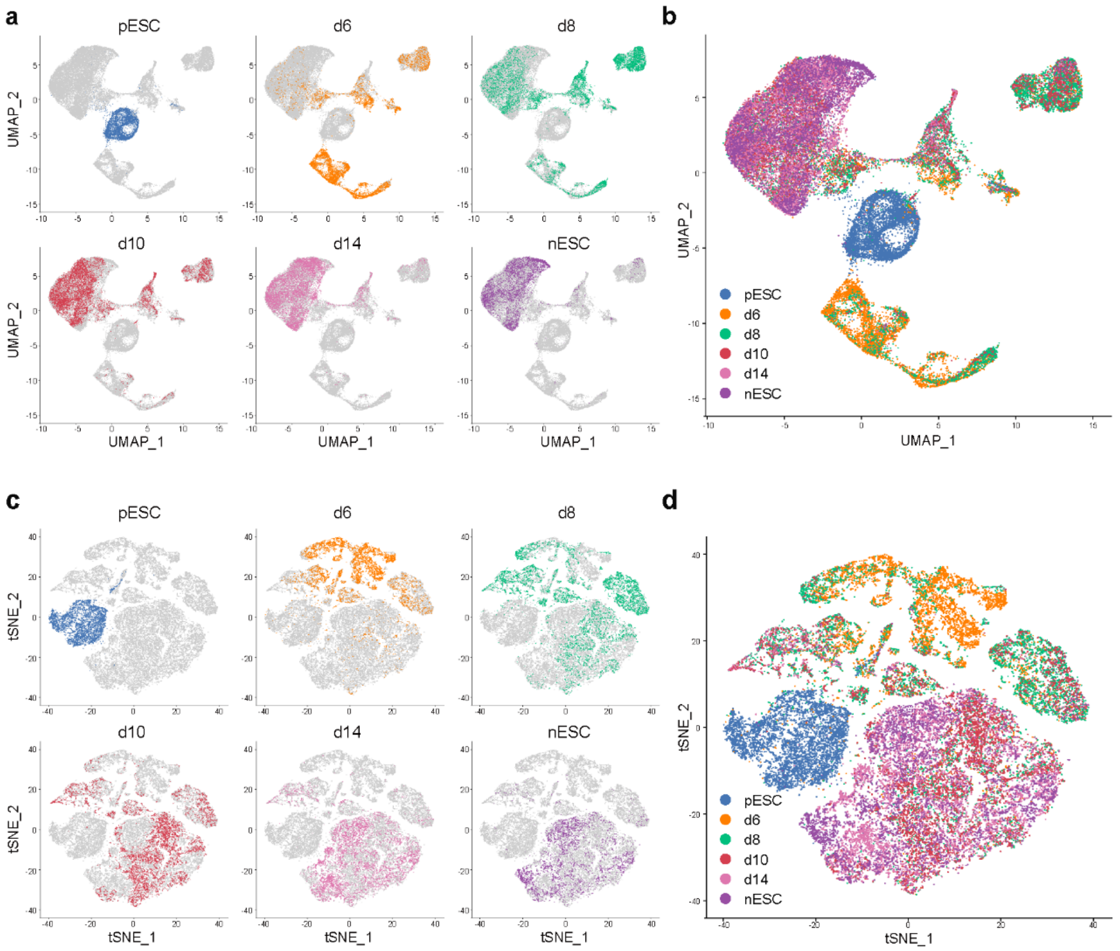

Supplementary Fig. 2 | scRNA-seq analysis during the primed-to-naive transition process.

a, UMAP plots highlighting cells within each time point or library.

b, UMAP plots of the integrated scRNA-seq datasets (a total of 38036 cells) with different libraries highlighted.

c, tSNE highlighting cells within each time point or library.

d, tSNE of the integrated scRNA-seq datasets (a total of 38036 cells) with different libraries highlighted.

Supplementary Fig. 3

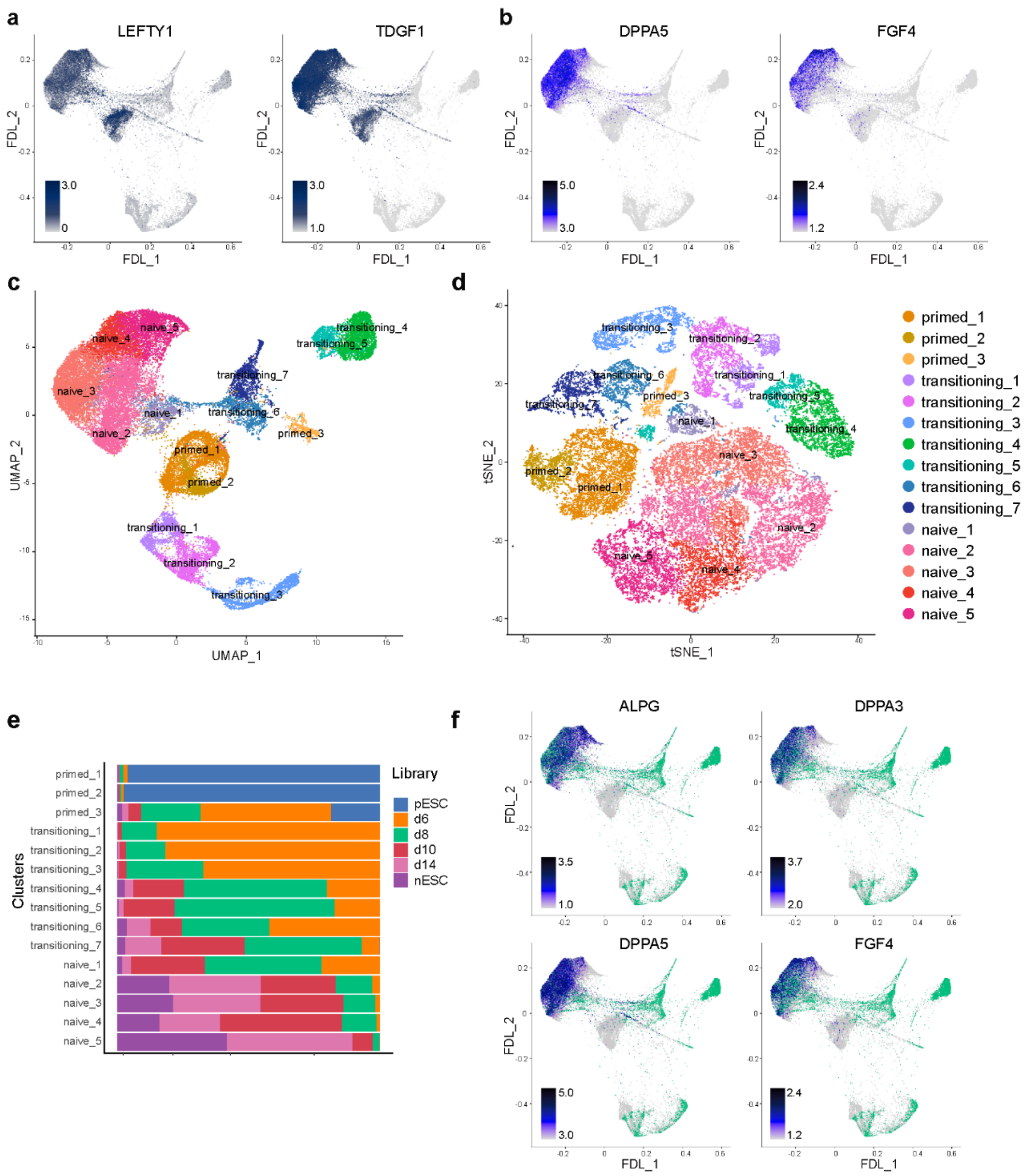

Supplementary Fig. 3 | Characterization of cell clusters.

a-b, Expression of marker genes associated with shared pluripotency, LEFTY1, TDGF1 (a, gray-blue); naive pluripotency, DPPA5, FGF4 (b, blue) on FDL.

c-d, Cell clustering projection on UMAP (c) and tSNE (d) dimensionality reduction, total 15 clusters.

e, Bar plot showing different libraries proportions of cell clusters in Fig.2f. Source data are provided as a Source Data file.

f, Expression of marker genes (blue) associated with naive pluripotency (ALPG, DPPA3, DPPA5, FGF4) on FDL with day 8 library (green) highlighted.

Supplementary Fig. 4

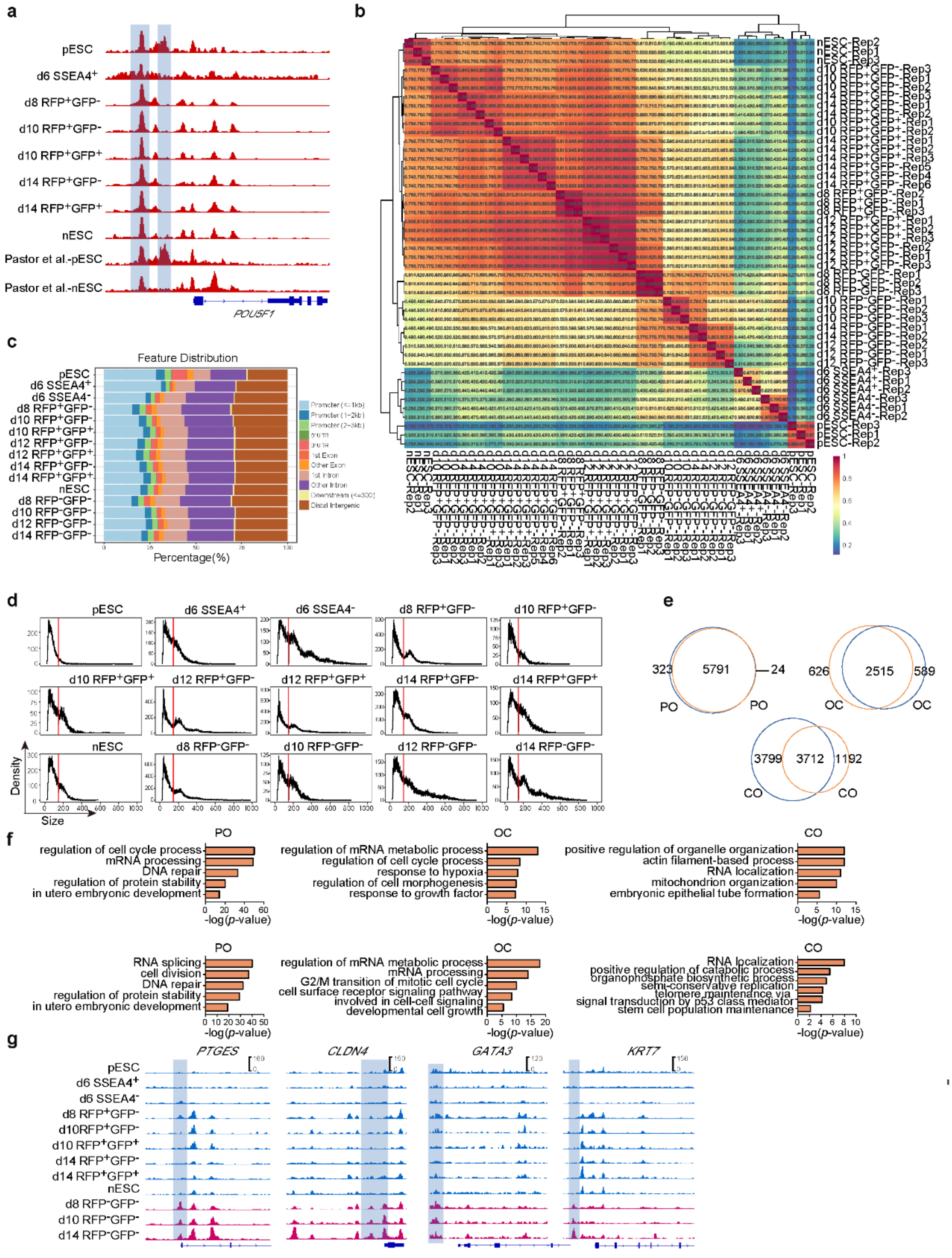

Supplementary Fig. 4 | Quality control for ATAC-seq datasets.

a, Chromatin landscape of POU5F1 with two putative enhancers, distal enhancer (DE) and proximal enhancer (PE) highlighted.

b, Heatmap representing the Pearson correlation coefficients among the ATAC-seq datasets.

c, The annotation of ATAC-seq peaks for each sample showing the feature distribution on the genome. Replicates ($n \geq 2$) with high correlation (> 0.8) were merged into one sample.

d, Density plots showing the length distribution of ATAC-seq peaks. NFR regions were defined (size < 147bp, red line).

e, Venn diagram to show the overlapping of gene loci between the two categories (red for Fig. 3b and blue for Fig. 3c) in PO, OC and CO groups, respectively.

f, Gene ontology (GO) analysis of genes with promoter regions (TSS±1kb) overlapped with ATAC-seq peaks for each PO, CO, or OC group in Fig. 3b-c. Upper panels corresponding to Fig. 3b, lower panels corresponding to Fig. 3c.

g, Representative ATAC-seq tracks on TE-specific gene loci for the CO peaks during the primed-to-naive transition.

Supplementary Fig. 5

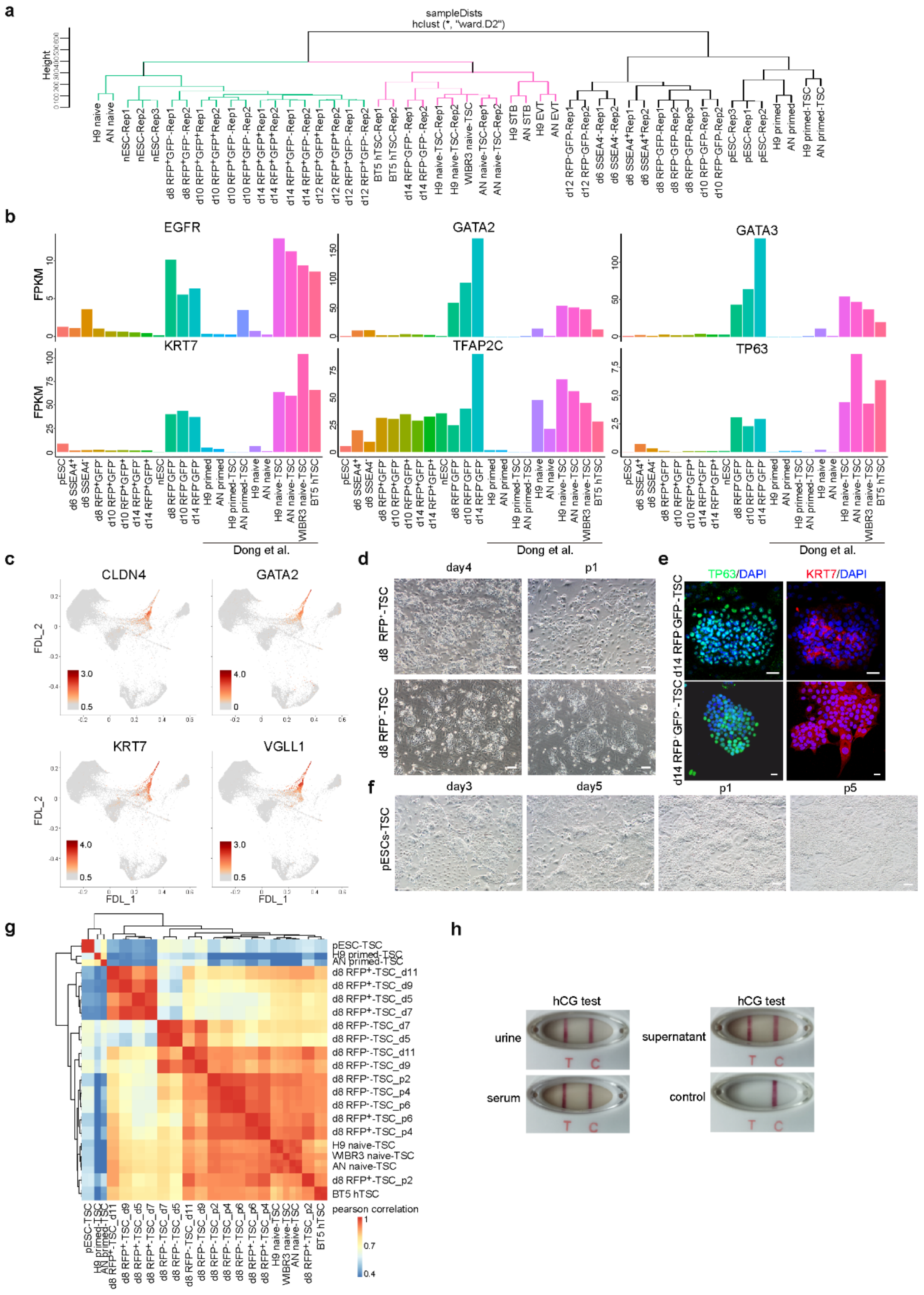

Supplementary Fig. 5 | Derivation of TSCs from the primed-to-naive transitioning intermediates with TE signatures.

a, Dendrogram showing the clustering distance among RNA-seq samples. Samples with naive pluripotency signature (green) and TSC signature (pink) were highlighted.

b, Bar plots showing the expression of representative TSC/TE marker genes among samples. Source data are provided as a Source Data file.

c, Expression of TSC/TE marker genes (CLDN4, GATA2, KRT7, VGLL1) on FDL.

d, Morphological changes during TSC induction from day 8-RFP^{+} and -RFP^{-} cells. Scale bars, 50 μm. Representative images from n = 5.

e, TP63 and KRT7 immunostaining of TSCs derived from day 14-RFP^{+} and day 14-RFP^{-} cells respectively during the primed-to-naive transition. Scale bars, 20 μm. Representative images from n = 3.

f, Morphological changes during TSC induction from pESCs. Scale bars, 50 μm. Representative images from n = 5.

g, Heatmap to indicate the Pearson correlation coefficients among the bulk RNA-seq datasets from the transitioning intermediates-derived TSCs with published RNA-seq datasets[33].

h, Representative positive results for hCG pregnancy test from urine samples, serum samples, and ST cell culture supernatant collected from day 8-RFP^{+} intermediates derived-TSCs. Source data are provided as a Source Data file.

Supplementary Fig. 6

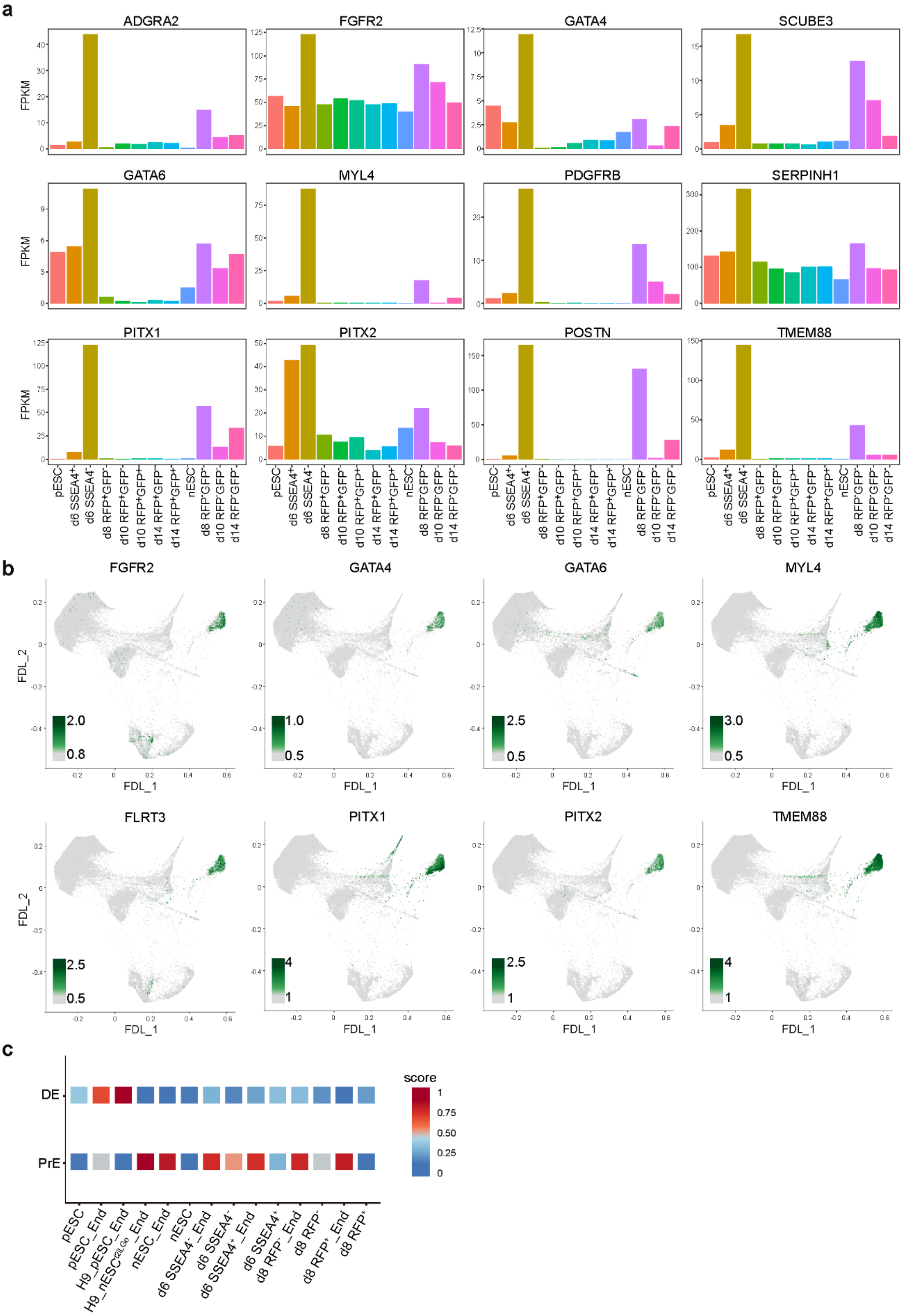

Supplementary Fig. 6 | Transcriptional profiling of cells at day 6 during the primed-to-naive transition process.

a, Bar plots showing the expression (FPKM) of representative PrE-specific genes during the primed-to-naive transition process. Source data are provided as a Source Data file.

b, Expression of representative PrE marker genes on FDL.

c, PrE and DE signature scores of the published endoderm cell lines, the primed-to-naive transitioning intermediates and endoderm cell lines derived from day6-SSEA4^{-} cells, day 6-SSEA4^{+} cells, day8 RFP^{-} cells, day8 RFP^{+} cells, pESCs, nESCs.

Supplementary Fig. 7

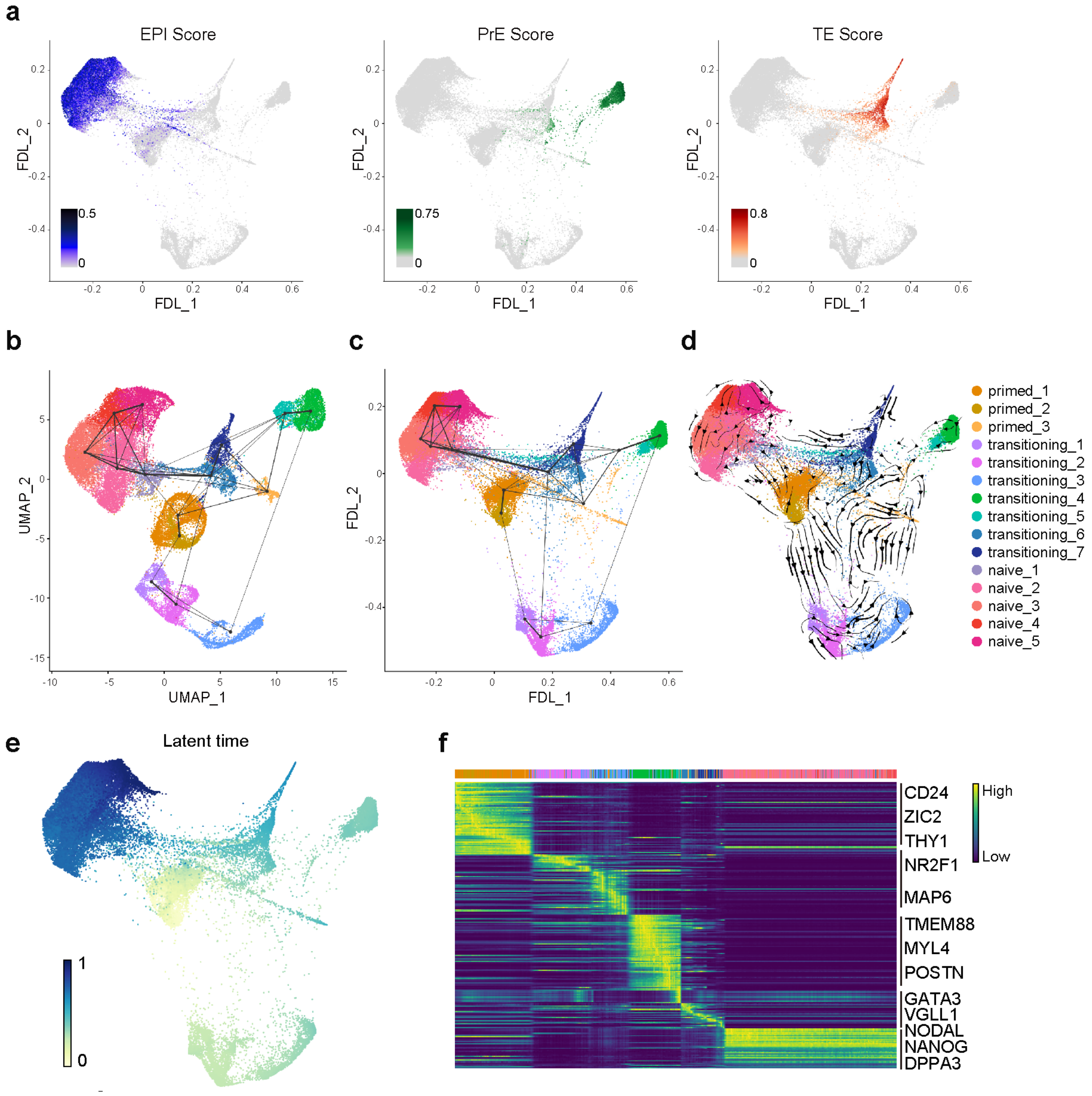

Supplementary Fig. 7 | Trajectory inference during the primed-to-naive transition process.

a, Per-cell expression score for EPI, TE, and PE signatures on FDL.

b, PAGA trajectory inference applied onto cell clusters in UMAP plots.

c, PAGA trajectory inference applied onto cell clusters on FDL.

d, RNA velocity applied onto cell clusters on FDL.

e, Latent time inference on FDL.

f, Heatmap showing latent time values of different clusters and transcriptional dynamics.
