# Supplementary material for: Cell fate roadmap of human primed-to-naive transition reveals preimplantation cell lineage signatures
Source: Nat Commun. 2022 Jun 7;13:3147. doi: 10.1038/s41467-022-30924-1 (PMC9174176; doi:10.1038/s41467-022-30924-1)
Supplement: Supplementary file 2 — Description of Additional Supplementary Files [file 41467_2022_30924_MOESM2_ESM.pdf]

File name: Supplementary Data 1

Description: The FPKM information of Bulk RNA-seq samples during primed-to-naive transition process. Information regarding K-means clustering of Bulk RNA-seq on gene expression and differential analysis in subpopulations of cells at day 6 and 8.

File name: Supplementary Data 2

Description: Gene lists for human epiblast-specific genes, primed-specific genes, TE, EPI, PrE, DE and TSC signatures used in this study.

File name: Supplementary Data 3

Description: ATAC-seq peak annotation in CAD for cells towards naive pluripotency.

File name: Supplementary Data 4

Description: ATAC-seq peak annotation in CAD for transitioning intermediates remaining RFP-negative.

File name: Supplementary Data 5

Description: The FPKM information of Bulk RNA-seq samples during TSC derivation from RFP+ and RFP- intermediates and prolonged 5iLAF induction process toward naive pluripotency.
