# Supplementary material for: Cell fate roadmap of human primed-to-naive transition reveals preimplantation cell lineage signatures
Source: Nat Commun. 2022 Jun 7;13:3147. doi: 10.1038/s41467-022-30924-1 (PMC9174176; doi:10.1038/s41467-022-30924-1)
Supplement: Supplementary file 8 — Reporting Summary [file 41467_2022_30924_MOESM8_ESM.pdf]

nature portfolio

Corresponding author(s): Shaorong Gao, Yixuan Wang

Last updated by author(s): May 4, 2022

Reporting Summary

Nature Portfolio wishes to improve the reproducibility of the work that we publish. This form provides structure for consistency and transparency in reporting. For further information on Nature Portfolio policies, see our Editorial Policies and the Editorial Policy Checklist.

Statistics

For all statistical analyses, confirm that the following items are present in the figure legend, table legend, main text, or Methods section.

n/a	Confirmed	
☐	☒	The exact sample size (*n*) for each experimental group/condition, given as a discrete number and unit of measurement
☐	☒	A statement on whether measurements were taken from distinct samples or whether the same sample was measured repeatedly
☐	☒	The statistical test(s) used AND whether they are one- or two-sided<br>*Only common tests should be described solely by name; describe more complex techniques in the Methods section.*
☒	☐	A description of all covariates tested
☐	☒	A description of any assumptions or corrections, such as tests of normality and adjustment for multiple comparisons
☐	☒	A full description of the statistical parameters including central tendency (e.g. means) or other basic estimates (e.g. regression coefficient) AND variation (e.g. standard deviation) or associated estimates of uncertainty (e.g. confidence intervals)
☐	☒	For null hypothesis testing, the test statistic (e.g. *F*, *t*, *r*) with confidence intervals, effect sizes, degrees of freedom and *P* value noted<br>*Give P values as exact values whenever suitable.*
☐	☒	For Bayesian analysis, information on the choice of priors and Markov chain Monte Carlo settings
☒	☐	For hierarchical and complex designs, identification of the appropriate level for tests and full reporting of outcomes
☐	☒	Estimates of effect sizes (e.g. Cohen's *d*, Pearson's *r*), indicating how they were calculated

Our web collection on statistics for biologists contains articles on many of the points above.

Software and code

Policy information about availability of computer code

Data collection	Microscopy images were taken using Zeiss LSM880 Microsystems. The flow cytometry data were collected in Cytoflex S (beckman coulter) and MoFlo XDP cell sorter (Beckman Coulter). RNA-sequencing, ATAC-sequencing and 10× Genomics scRNA-sequencing were performed at Berry Genomics Corporation.
Data analysis	Images were processed with the ZFN software (Zeiss LSM880), Flow cytometry data were processed using Flow Jo software (V10.0). Statistical analyses were carried out using the Prism software (GraphPad V8.0) .<br>RNA-seq raw reads were processed by Trim_galore (version 0.6.6) to remove adapters and low-quality reads with the default parameters. Bulk RNA-seq reads were then aligned to the human genome (hg38) using STAR (STAR_2.5.2b) with the default parameters excepting using the "--outSAMattrIHstart 0", "--outSAMstrandField intronMotif", "--outFilterIntronMotifs RemoveNoncanonical", "--outFilterMismatchNmax 999", "--outFilterMismatchNoverReadLmax 0.04", "--quantMode GeneCounts", "--twopassMode Basic" parameters. Expression levels of all Refseq genes for samples were quantified to FPKM using Stringtie (version 2.1.4).<br>The ATAC-seq sequencing data were pre-processed by Trim_galore (version 0.6.6) to remove adapters and low-quality reads with the default parameters. All the cleaned reads were aligned to the human genome assembly (hg38) using bowtie2 (version 2.4.1) with the default parameters except the following options: -X 2000 --no-unal --very-sensitive. Reads mapping to mitochondrial DNA were discarded using "grep –v chrM". Only high quality mapped reads and concordantly aligned pairs were retained using samtools (view –q 30 -f 2). Motif analysis was performed using HOMER (v.4.11.1).<br>The 10× Genomics single-cell data were pre-processed using the Cell Ranger pipeline (v.4.0.0) with default parameters to generate the expression matrix. For quality control, all cutoffs were determined after investigating the distributions of each variable. Cells with a low number of expressed genes (nFeature) or extremely high counts (nCount) or a high percentage of mitochondrial genes (pctMT) were discarded. Cutoffs of nFeature >2,500, 1,000 < nCount < 100,000 and pctMT < 10 were applied to retain cells. Genes were also filtered if not present in at least 10 cells with at least 1 read each. Ribosomal genes were also removed for downstream analysis. Seurat v.3 (v.3.2.3) was utilized to integrate datasets,reduce dimension and visualization. Scanpy package (v.1.7.2) was utilized to compute ForceAtlas 2 layout and PAGA. Velocyto (v.0.17.17) and scVelo (v.0.2.4) were utilized to perform RNA velocity analysis.

Scripts can be found at https://github.com/zftu/Human-primed-to-naive-transition-analysis.

For manuscripts utilizing custom algorithms or software that are central to the research but not yet described in published literature, software must be made available to editors and reviewers. We strongly encourage code deposition in a community repository (e.g. GitHub). See the Nature Portfolio guidelines for submitting code & software for further information.

Data

Policy information about availability of data

All manuscripts must include a data availability statement. This statement should provide the following information, where applicable:

- Accession codes, unique identifiers, or web links for publicly available datasets
- A description of any restrictions on data availability
- For clinical datasets or third party data, please ensure that the statement adheres to our policy

The main data supporting the findings of this study are available within the article and its Supplementary Information files. The bulk RNA-seq datasets, scRNA-seq datasets and ATAC-seq datasets generated in this study are available at GEO: GSE173756 [https://www.ncbi.nlm.nih.gov/geo/query/acc.cgi?acc=GSE173756] and GSE174771 [https://www.ncbi.nlm.nih.gov/geo/query/acc.cgi?acc=GSE174771]. The accession number for the RNA-seq data of human embryos is GSE36552 [https://www.ncbi.nlm.nih.gov/geo/query/acc.cgi?acc=GSE36552]. The accession numbers for the RNA-seq data of published TSC cell lines is GSE138762 [https://www.ncbi.nlm.nih.gov/geo/query/acc.cgi?acc=GSE138762]. The accession number for the RNA-seq data of End cell lines is GSE138012 [https://www.ncbi.nlm.nih.gov/geo/query/acc.cgi?acc=GSE138012]. There is no restrictions on data availability.

Field-specific reporting

Please select the one below that is the best fit for your research. If you are not sure, read the appropriate sections before making your selection.

☒ Life sciences ☐ Behavioural & social sciences ☐ Ecological, evolutionary & environmental sciences

For a reference copy of the document with all sections, see nature.com/documents/nr-reporting-summary-flat.pdf

Life sciences study design

All studies must disclose on these points even when the disclosure is negative.

Sample size	500ng RNA per reaction for mRNA-sequencing (follow KAPA stranded mRNA-Seq kit (KAPA)), 50,000 cells per reaction for ATAC-seq (Wu, J. et al.Nature,2016;TD501-TD503, Vazyme) and 100,000 cells per reaction for 10x Genomics scRNA-seq (according to the manufacturer's instructions). According to standards in the field and the experimental experience and knowledge to choose an adequate pool for FACS, microscopy experiments. No statistical method was used to predetermine sample size.
Data exclusions	No data were excluded from the analyses.
Replication	For each representative image, experiments were performed at least three times with similar results. RNA-seq was performed 2-4 times with the number of replications. ATAC-seq were performed 3-6 times with the number of replications. 10× genomics scRNA-seq data, libraries at pES (n = 1), day 6 (n = 1), day 8 (n = 1), day 10 (n = 1), day 14 (n = 1), nES (n = 1) were generated. Flow cytometry analysis and immunostaining were performed at least three times.<br>All attempts at replication were successful.
Randomization	No randomization methods were utilized except for experiments using animals. Animals used for experiments were randomly allocated.
Blinding	The investigators were not blinded during data collection and analysis. We did not consider blinding required in this study.

Reporting for specific materials, systems and methods

We require information from authors about some types of materials, experimental systems and methods used in many studies. Here, indicate whether each material, system or method listed is relevant to your study. If you are not sure if a list item applies to your research, read the appropriate section before selecting a response.

Materials & experimental systems

n/a	Involved in the study	
☐	☒	Antibodies
☐	☒	Eukaryotic cell lines
☒	☐	Palaeontology and archaeology
☐	☒	Animals and other organisms
☒	☐	Human research participants
☒	☐	Clinical data
☒	☐	Dual use research of concern

Methods

n/a	Involved in the study	
☒	☐	ChIP-seq
☐	☒	Flow cytometry
☒	☐	MRI-based neuroimaging

Antibodies

Antibodies used

Alexa Fluor647 anti-human SSEA4 (BioLegend, Cat#330407,RRID: AB_1089201,1:50 dilution)
anti-HLA-G (Abcam, Cat#ab7759, RRID: AB_306053,Lot GR3262011-5,1:500 dilution)
Anti-CGB(HCG) (Abcam, Cat#ab131170, RRID:ab_11156864,1:500 dilution)
APC anti KRT7 (Abcam, Cat#ab192077,Lot GR3214132-7,1:500 dilution)
anti-TP63 (Cell Signaling, Cat#13109T, Lot 3,1:800 dilution)
anti-SDC1 (Abcam, Cat#ab181789,Lot GR317857,1:500 dilution)
Goat anti-GATA6 (R&D Systems,Cat#AF1700,Lot KWT0418111,1:200 dilution)
Anti-SUSD2 Mouse Monoclonal Antibody (APC) (BioLegend,Cat#327408,clone:W5C5,RRID:AB_2561888,1:50 dilution)
4',6-diamidino-2-phenylindole (Sigma,Cat#D8417,1:1000 dilution)

Alexa Fluor® 488 Donkey Anti-Rabbit IgG (H+L) Antibody (Invitrogen,Cat#A-21206,RRID:AB_2535792,1:500 dilution)
Alexa Fluor® 488 Donkey Anti-Mouse IgG (H+L) Antibody (Thermo Scientific,Cat#A-21202,RRID:AB_2536180,1:500 dilution)
Alexa Fluor® 594 Donkey Anti-Rabbit IgG (H+L) Antibody (Fisher Scientific,Cat#A-21207, RRID:AB_141637,1:500 dilution)
Alexa Fluor® 594 Donkey Anti-Mouse IgG (H+L) Antibody (Fisher Scientific,Cat#A-21203, RRID:AB_141633,1:500 dilution)
Alexa Fluor® 647 Donkey Anti-Rabbit IgG (H+L) Antibody (Thermo Scientific,Cat#A-31573,RRID:AB_2536183,1:500 dilution)
Alexa Fluor® 647 Donkey Anti-Mouse IgG (H+L) Antibody (Thermo Scientific,Cat#A-31571,RRID:AB_162542,1:500 dilution)

Validation

-Alexa Fluor647 anti-human SSEA4 (BioLegend, Cat#330407,RRID: AB_1089201,1:50 dilution): The antibody guarantee covers the use of the antibody for IF applications. The antibody has been referenced in 22 publications. https://www.biolegend.com/nl-be/products/alexa-fluor-647-anti-human-ssea-4-antibody-4823
-anti-HLA-G (Abcam, Cat#ab7759, RRID: AB_306053,1:500 dilution):The antibody guarantee covers the use of the antibody for IF applications. The antibody has been referenced in 21 publications. https://www.abcam.com/hla-g-antibody-mem-g1-ab7759.html
-Anti-CGB(HCG) (Abcam, Cat#ab131170, RRID:ab_11156864,1:500 dilution): The antibody guarantee covers the use of the antibody for IF applications. The antibody has been referenced in 6 publications. https://www.abcam.com/hcg-beta-antibody-ab53087.html
-APC anti KRT7 (Abcam, Cat#ab192077,1:500 dilution):The antibody guarantee covers the use of the antibody for IF applications. https://www.abcam.com/alexa-fluor-647-cytokeratin-7-antibody-epr1619y-cytoskeleton-marker-ab192077.html
-anti-TP63 (Cell Signaling, Cat#13109T,1:800 dilution):The antibody guarantee covers the use of the antibody for IF applications. The antibody has been referenced in 50 publications. https://www.cellsignal.com/products/primary-antibodies/p63-a-d2k8x-xp-rabbit-mab/13109
-anti-SDC1 (Abcam, Cat#181789,1:500 dilution):The antibody guarantee covers the use of the antibody for IF applications. The antibody has been referenced in 4 publications. https://www.abcam.com/syndecan-1-antibody-1a3h4-ab181789.html
oat anti-GATA6 (R&D Systems,Cat#AF1700,Lot KWT0418111,1:200 dilution):The antibody guarantee covers the use of the antibody for IF applications. The antibody has been referenced in 44 publications. https://www.rndsystems.com/products/human-gata-6-antibody_af1700
Anti-SUSD2 Mouse Monoclonal Antibody (APC) (BioLegend,Cat#327408,1:50 dilution):The antibody guarantee covers the use of the antibody for IF applications. The antibody has been referenced in 2 publications. https://www.biolegend.com/en-us/products/apc-anti-human-susd2-antibody-8410
4',6-diamidino-2-phenylindole (Sigma,Cat#D8417,1:1000 dilution):The antibody guarantee covers the use of the antibody for IF applications. https://www.sigmaaldrich.com/US/en/product/sigma/d8417

-Alexa Fluor® 488 Donkey Anti-Rabbit IgG (H+L) Antibody (Invitrogen,Cat#A-21206,RRID:AB_2535792,1:500 dilution):The antibody guarantee covers the use of the antibody for IF applications. The antibody has been referenced in 4266 publications. https://www.thermofisher.com/antibody/product/Donkey-anti-Rabbit-IgG-H-L-Highly-Cross-Adsorbed-Secondary-Antibody-Polyclonal/A-21206
-Alexa Fluor® 488 Donkey Anti-Mouse IgG (H+L) Antibody (Thermo Scientific,Cat#A-21202,RRID:AB_2536180,1:500 dilution):The antibody guarantee covers the use of the antibody for IF applications. The antibody has been referenced in 3147 publications. https://www.thermofisher.com/antibody/product/Donkey-anti-Mouse-IgG-H-L-Highly-Cross-Adsorbed-Secondary-Antibody-Polyclonal/A-21202
-Alexa Fluor® 594 Donkey Anti-Rabbit IgG (H+L) Antibody (Fisher Scientific,Cat#A-21207, RRID:AB_141637,1:500 dilution):The antibody guarantee covers the use of the antibody for IF applications. The antibody has been referenced in 1606 publications. https://www.thermofisher.com/antibody/product/Donkey-anti-Rabbit-IgG-H-L-Highly-Cross-Adsorbed-Secondary-Antibody-Polyclonal/A-21207
-Alexa Fluor® 594 Donkey Anti-Mouse IgG (H+L) Antibody (Fisher Scientific,Cat#A-21203, RRID:AB_141633,1:500 dilution):The antibody guarantee covers the use of the antibody for IF applications. The antibody has been referenced in 1008 publications. https://www.thermofisher.com/antibody/product/Donkey-anti-Mouse-IgG-H-L-Highly-Cross-Adsorbed-Secondary-Antibody-Polyclonal/A-21203
-Alexa Fluor® 647 Donkey Anti-Rabbit IgG (H+L) Antibody (Thermo Scientific,Cat#A-31573,RRID:AB_2536183,1:500 dilution):The antibody guarantee covers the use of the antibody for IF applications. The antibody has been referenced in 1327 publications. https://www.thermofisher.com/antibody/product/Donkey-anti-Rabbit-IgG-H-L-Highly-Cross-Adsorbed-Secondary-Antibody-Polyclonal/A-31573
-Alexa Fluor® 647 Donkey Anti-Mouse IgG (H+L) Antibody (Thermo Scientific,Cat#A-31571,RRID:AB_162542,1:500 dilution):The antibody guarantee covers the use of the antibody for IF applications. The antibody has been referenced in 1140 publications. https://www.thermofisher.com/antibody/product/Donkey-anti-Mouse-IgG-H-L-Highly-Cross-Adsorbed-Secondary-Antibody-Polyclonal/A-31571

Eukaryotic cell lines

Policy information about cell lines

Cell line source(s)

293T (human embryonic kidney cells) were acquired from ATCC (CRL-3216). 5iLAF-cultured H9 hESCs (ALPG-promoter-RFP; OCT4-ΔPE-GFP) were reported previously (Bi et al, Cell Reports, 2020), H9 hESCs with OCT4-ΔPE-GFP reporter were kindly

provided by Haoyi Wang (Institute of Zoology, CAS).
TSCs were derived from the intermediates during primed-to-naive transition process, primed- TSCs as control. EVT and ST cells were differentiated from the corresponding TSCs. MEF cells were established from embryonic day 11.5-13.5 (E11.5-E13.5) by uterine dissection for individual embryos in our laboratory.

Authentication	For human pESCs and nESCs, we performed RNA-seq analysis, the transcriptomes of pESCs and the corresponding nESCs clustered with those in published datasets of conventional cultured PSCs and ICM, respectively. For TSCs, we performed RNA-seq analysis, the transcriptomes of TSC derived in our lab clustered with those in published datasets of TSCs.
Mycoplasma contamination	All cell lines were tested negative for mycoplasma contamination. None of the cell lines were contaminated. Please refer to methods section for details.
Commonly misidentified lines (See ICLAC register)	No commonly misidentified lines were used.

Animals and other organisms

Policy information about studies involving animals; ARRIVE guidelines recommended for reporting animal research

Laboratory animals	NOD/SCID IL-2R-gamma knockout mice, famale, 5-20 weeks of age were used. Mice were housed in 12-hr light/12-hr dark cycle 22.1–22.3 °C and 33–44% humidity
Wild animals	No wild animals were used.
Field-collected samples	No field-collected samples were used.
Ethics oversight	All experiments were performed in accordance with the University of Health Guide for the Care and Use of Laboratory Animals and were approved by the Biological Research Ethics Committee of Tongji University.

Note that full information on the approval of the study protocol must also be provided in the manuscript.

Flow Cytometry

Plots

Confirm that:

☒ The axis labels state the marker and fluorochrome used (e.g. CD4-FITC).

☒ The axis scales are clearly visible. Include numbers along axes only for bottom left plot of group (a 'group' is an analysis of identical markers).

☒ All plots are contour plots with outliers or pseudocolor plots.

☒ A numerical value for number of cells or percentage (with statistics) is provided.

Methodology

Sample preparation	Analysis or sorting of cells by flow cytometry was described in the Methods section. Briefly,cells were collected, washed with FACS buffer containing PBS supplemented with 2% FBS. After stained with antibodies, cells were washed and resuspended in FACS buffer.
Instrument	CytoFlex S (Beckman Coulter) for flow cytometry analysis and MoFlo Astrios 4 laser (Beckman Coulter) for cell sorting.
Software	CytExpert 2.3 (Beckman) was used to collect the flow cytometry data. Flowjo_V10 was used for analysis.
Cell population abundance	For analyses, over 100,000 cells were gated each time for analyze. See Method section details.
Gating strategy	Preliminary FSC/SSC gating was selected by the scatter plots of normal cell population, and gate boundaries were determined by the positive/negative control and based on experience.

☒ Tick this box to confirm that a figure exemplifying the gating strategy is provided in the Supplementary Information.
